# Pain and treatment outcomes after initiating methadone vs buprenorphine among medicare patients with opioid use disorder and comorbid chronic pain: A target trial emulation

**DOI:** 10.1371/journal.pmed.1004846

**Published:** 2026-03-26

**Authors:** Yu-Jung Jenny Wei, Almut G. Winterstein, Roger B. Fillingim, Stephan Schmidt, Siegfried Schmidt

**Affiliations:** 1 Division of Pharmaceutics and Pharmacology, College of Pharmacy, The Ohio State University, Columbus, Ohio, United States of America; 2 Department of Pharmaceutical Outcomes and Policy, College of Pharmacy, University of Florida, Gainesville, Florida, United States of America; 3 Center for Drug Evaluation and Safety, University of Florida, Gainesville, Florida, United States of America; 4 Department of Epidemiology, Colleges of Medicine and Public Health & Health Professions, University of Florida, Gainesville, Florida, United States of America; 5 Pain Research and Intervention Center of Excellence, University of Florida, Gainesville, Florida, United States of America; 6 Department of Pharmaceutics, College of Pharmacy, University of Florida, Gainesville, Florida, United States of America; 7 Department of Community Health and Family Medicine, College of Medicine, University of Florida, Gainesville, Florida, United States of America; University of New South Wales, AUSTRALIA

## Abstract

**Background:**

Methadone and buprenorphine, effective treatments for opioid use disorder (OUD), also provide analgesia for managing pain, which is commonly experienced by patients with OUD. Limited population-based evidence exists comparing pain-related and treatment outcomes for methadone versus buprenorphine among patients with OUD and comorbid pain. The study aims to examine pain-related and treatment outcomes among Medicare patients with comorbid pain and OUD who initiated methadone or buprenorphine.

**Methods and findings:**

We conducted a retrospective cohort study with target trial emulation using the 100% Medicare data from 2020 to 2023. Participants included patients with comorbid chronic pain and OUD who initiated methadone or buprenorphine. The key dependent variables were pain-related outcomes that included hospitalization and emergency department (ED) visit due to pain, and treatment outcomes that included opioid overdose and all-cause mortality. Outcomes were assessed 1 year following treatment initiation. Intention-to-treat and per-protocol analyses were conducted to estimate incidence rate ratios (IRRs) for pain-related outcomes and opioid overdose and hazard ratios (HRs) for all-cause mortality. For each outcome, we also calculated the adjusted risk difference (aRD) between the methadone and buprenorphine groups. We identified 49,727 eligible Medicare patients (mean [SD] age, 59.0 [11.6] years; 24,538 [49.3%] female and 25,189 [50.7%] male). Of the identified patients, 16,174 (32.5%) initiated methadone solely administered at opioid treatment programs, and 33,553 (67.5%) initiated buprenorphine primarily prescribed at office-based clinics. Compared with buprenorphine, initiation of methadone was associated with lower adjusted incidence rates of pain-related hospitalization (IRR, 0.64 (95% CI [0.58, 0.70]; *P* < .001); aRD, −7.2 (95% CI [−8.8 to −5.7]) per 1,000 person-years) and ED visit (IRR, 0.87 (95% CI [0.82, 0.92]; *P* < .001); aRD, −10.2 (95% CI [−14.4, −5.9]) per 1,000 person-years) in per-protocol analyses, with no difference in opioid overdose (IRR, 1.02 (95% CI [0.93,1.10]; *P* = .72); aRD, 0.33 (95% CI [−1.5, 2.1]) per 1,000 person-years) and all-cause mortality (HR, 1.06 (95% CI, [0.81–1.39]; *P* = .66); aRD, 1.1 (95% CI [−1.3, 1.0]) per 1,000 person-years) rates. Similar results were observed in intention-to-treat analyses. Main study limitations included unmeasured confounders and limited generalizability.

**Conclusions:**

This population-based cohort study of Medicare patients with comorbid chronic pain and OUD found that methadone administered at opioid treatment programs is associated with reduced hospitalizations and ED visits for pain-related visits while offering treatment outcomes similar to buprenorphine primarily prescribed at office-based clinics. The favorable pain-related outcomes in patients with methadone should be interpreted with caution, as the finding may reflect differences in the underlying patient population, treatment dosing practices, pharmacological properties, and treatment practice settings, which cannot be measured in Medicare data and merit further investigations.

## Author summary

**Why was this study done?:**

- Methadone and buprenorphine, effective treatments for opioid use disorder (OUD), also provide analgesia for managing pain, which is commonly experienced by patients with OUD.
- While studies have examined pain outcomes among patients prescribed medications for OUD, their quality of evidence is low, with small sample sizes, and they are mostly restricted to patients in inpatient or peri- and post-operative settings.
- Limited studies have also compared treatment outcomes between Medicare patients prescribed different types of medications for OUD after Medicare’s expansion covering methadone in 2020.

**What did the researchers do and find?:**

- The analysis assessed pain-related and treatment outcomes during the follow-up (up to 1 year) after the initiation of methadone or buprenorphine among 49,727 Medicare patients with OUD and comorbid chronic pain.
- We found lower incidence rates for pain-related hospitalizations and pain-related emergency room visits, with no difference in opioid overdose and all-cause mortality rates within 1 year following initiation of methadone compared with buprenorphine.

**What do these findings mean?:**

- Methadone administered at opioid treatment programs may reduce hospitalizations and emergency room visits for pain-related visits while offering treatment outcomes similar to buprenorphine, primarily prescribed at office-based clinics.
- The favorable pain-related outcomes in patients with methadone versus buprenorphine should be interpreted with caution, as the finding may reflect differences in the underlying patient population, treatment dosing practices, pharmacological properties, and treatment practice settings, which cannot be measured in Medicare data and merit further investigations.
- The study findings are only generalizable to Medicare patients with chronic pain and receipt of methadone or buprenorphine for OUD and are limited by not fully accounting for confounders, particularly those that cannot be measured in Medicare data.

## Abstract 3

In a retrospective cohort study with a target emulation trial design, Wei and colleagues investigate pain-related outcomes, measured as hospitalization and emergency department visits, after methadone or buprenorphine initiation in Medicare beneficiaries treated for opioid use disorder with comorbid chronic pain.

## Introduction

Opioid use disorder (OUD), a chronic health condition caused by dependence of prescription opioids or illicit opioids, has been a major public health concern in many countries [1]. In the United States (US), OUD has increased over the years in populations, including those enrolled in Medicare, which comprises individuals 65 years or older and individuals with disabilities 64 years or younger. The number of Medicare beneficiaries with a diagnosis of OUD soared from 0.03 million in 2013 [2] to 2.0 million in 2018 [3], and then followed a steady decline to 1.1 million in 2022 [4], which remained higher than in the early 2010s [2]. Approximately half (51.1%) of Medicare fee-for-service beneficiaries with OUD are under the age of 65 years [3]. To address the increase in OUD in Medicare populations, the Centers for Medicare and Medicaid Services, the single largest US health insurer, provides coverage of Food and Drug Administration (FDA)-approved medications for OUD, including methadone (starting January 1, 2020), buprenorphine, and naltrexone, prescribed either at government-certified opioid treatment programs (OTPs) or office-based clinics, and covers the cost of OUD services and medications for eligible beneficiaries [5, 6]. Medicare’s new coverage for methadone in 2020 increased its prescribing from 0.98 per 1,000 patients with OUD in 2020 to 4.71 in 2022 [7]. A similar increase in buprenorphine prescribing was also observed from 4.64 per 1,000 Medicare patients with OUD in 2019 to 7.45 in 2022 [7].

With the increase in Medicare patients with OUD, an emerging clinical concern is how to effectively manage co-occurring comorbid pain [8–10], which includes over two-thirds (76.1%) of the affected patients [3]. Methadone and buprenorphine not only are clinically effective and recommended treatments for OUD but also provide analgesia and have an FDA-approved indication for pain management [11, 12]. Methadone, a full µ-opioid receptor agonist with high intrinsic activity, produces increasing analgesia with increasing doses, while buprenorphine, a partial µ-opioid receptor opioid with low intrinsic activity, provides less analgesic effect that may reach its maximum at increasing doses [11, 12], although the ceiling effect on buprenorphine analgesic properties remains unclear [13]. On the other hand, buprenorphine is recommended by clinical experts in management of both pain and OUD [14], particularly in inpatient and perioperative settings [8, 15] given that buprenorphine is less federally regulated [11] and has a superior safety profile with limited respiratory depression due to a drug ceiling effect at high doses [11] and has lower risk of illicit drug use and diversion compared with methadone [16]. Also, buprenorphine demonstrated superior efficacy in managing acute post-operative pain compared with full agonist opioids in a recent review of 58 randomized controlled trials [17]. In addition to the pharmacological difference between methadone and buprenorphine, these two OUD treatments are offered in different practice settings, with methadone administered daily (except Sunday) at OTPs that are highly regulated and structured [18], whereas buprenorphine is mostly prescribed at office-based clinics with less stringent regulations and greater convenience in access to treatment [19].

Limited data exist regarding comparative evidence for pain-related outcomes between methadone and buprenorphine [10, 20, 21], two prevalently prescribed medications for OUD for Medicare patients with OUD [7]. Although studies have examined pain outcomes among patients prescribed medications for OUD, their quality of evidence is low, with small sample sizes, and they are mostly restricted to patients in inpatient or peri- and post-operative settings [10, 20-22]. Limited studies have compared treatment outcomes between Medicare patients prescribed different types of medications for OUD after Medicare’s expansion covering methadone in 2020. The few existing studies involving Medicare populations primarily compared drug overdose between patients with versus without receipt of medications for OUD before and after the COVID-19 pandemic period (up to early 2021) [23, 24].

To address these research gaps, we conducted a population-based cohort study using a target trial emulation design to compare pain-related and treatment outcomes (i.e., opioid overdose and all-cause mortality) among Medicare patients with comorbid chronic pain and OUD who initiated methadone or buprenorphine treatment between 2020 and 2023. We hypothesized that initiation of methadone versus buprenorphine was associated with pain-related and treatment outcomes.

## Methods

### Data sources

We used a 100% Medicare sample with administrative billing records of Medicare Advantage and Fee-for-service beneficiaries, starting January 1, 2020, and ending December 31, 2022, for Medicare Advantage enrollees and December 31, 2023, for Fee-for-service enrollees. We used Medicare Part B (for Fee-for-service enrollees) and Part C data (for Medicare Advantage enrollees) to capture medications for OUD covered under bundled payments and dispensed at OTP facilities or office-based clinics. We used Part D prescription data (for both Fee-for-service and Medicare Advantage enrollees) to capture prescriptions of medications for OUD dispensed at pharmacies. The Ohio State University’s Institutional Review Board approved this study and waived the informed consent requirement because the data were deidentified. This study followed the Transparent Reporting of Observational Studies Emulating a Target Trial (TARGET) guideline for cohort studies (S1 TARGET Checklist).

### Study design

Using a retrospective cohort study design, we emulated a hypothetical target trial [25] to compare outcomes of initiating methadone versus buprenorphine among Medicare patients. We summarized the key elements of the target trial in Table 1 and detailed them as follows.

**Table 1:** Specification and emulation of a target trial comparing outcomes of methadone vs. buprenorphine among medicare patients.

Protocol element	Target trial specification	Trial emulation using 100% Medicare sample
Eligibility criteria	Medicare patients initiating methadone or buprenorphine for treatment of OUD between July 1, 2020, and December 31, 2023 Inclusion criteria Diagnosis of opioid use disorder^a^ Diagnosis of chronic pain^a^ Initiated buprenorphine with a formulation indicated for OUD dispensed from pharmacies, office-based clinics, or OTPs Initiated methadone dispensed from OTPs only Exclusion criteria^a^: No continuous Medicare enrollment Diagnosis of cancer, hospice, or palliative care Hospital or skilled nursing facility stay ^a^ 6 months before treatment initiation	Same as in the target trial
Treatment strategies	Initiation of methadone vs. buprenorphine for treatment of OUD; index date = date of treatment initiation	Same as in the target trial
Treatment assignment	Individuals randomly assigned to one of the treatment strategies at index date Neither patients nor providers blinded to intervention	Randomization of treatment assignment was emulated using IPTW to account for baseline characteristics Neither patients nor providers blinded to intervention
Follow-up	ITT analysis: starts index date and ends at 1-year follow-up, death, Medicare disenrollment, or study end, whichever occurs first Per-protocol analysis: patients additionally censored when discontinuing assigned treatment	Same as in the target trial
Outcomes	Pain outcomes included pain-related hospitalization and ED visit; treatment outcomes included opioid overdose and all-cause mortality	Same as in the target trial
Causal contrasts	Per-protocol effect ITT effect	Same as in the target trial
Statistical analysis	ITT analysis: assessed balance on baseline covariates Per-protocol analysis: potential selection bias additionally adjusted via IPCW due to treatment discontinuation	ITT analysis: adjusted baseline covariates via IPTW Per-protocol analysis: adjusted baseline covariates via IPTW and potential selection bias via IPCW due to treatment discontinuation

Abbreviations: ED, emergency department; OTP, opioid treatment program; OUD, opioid use disorder; IPCW, inverse probability of censoring weighting; IPTW, inverse probability of treatment weighting; ITT, intention-to-treat.

### Eligibility criteria

**Fig 1:**
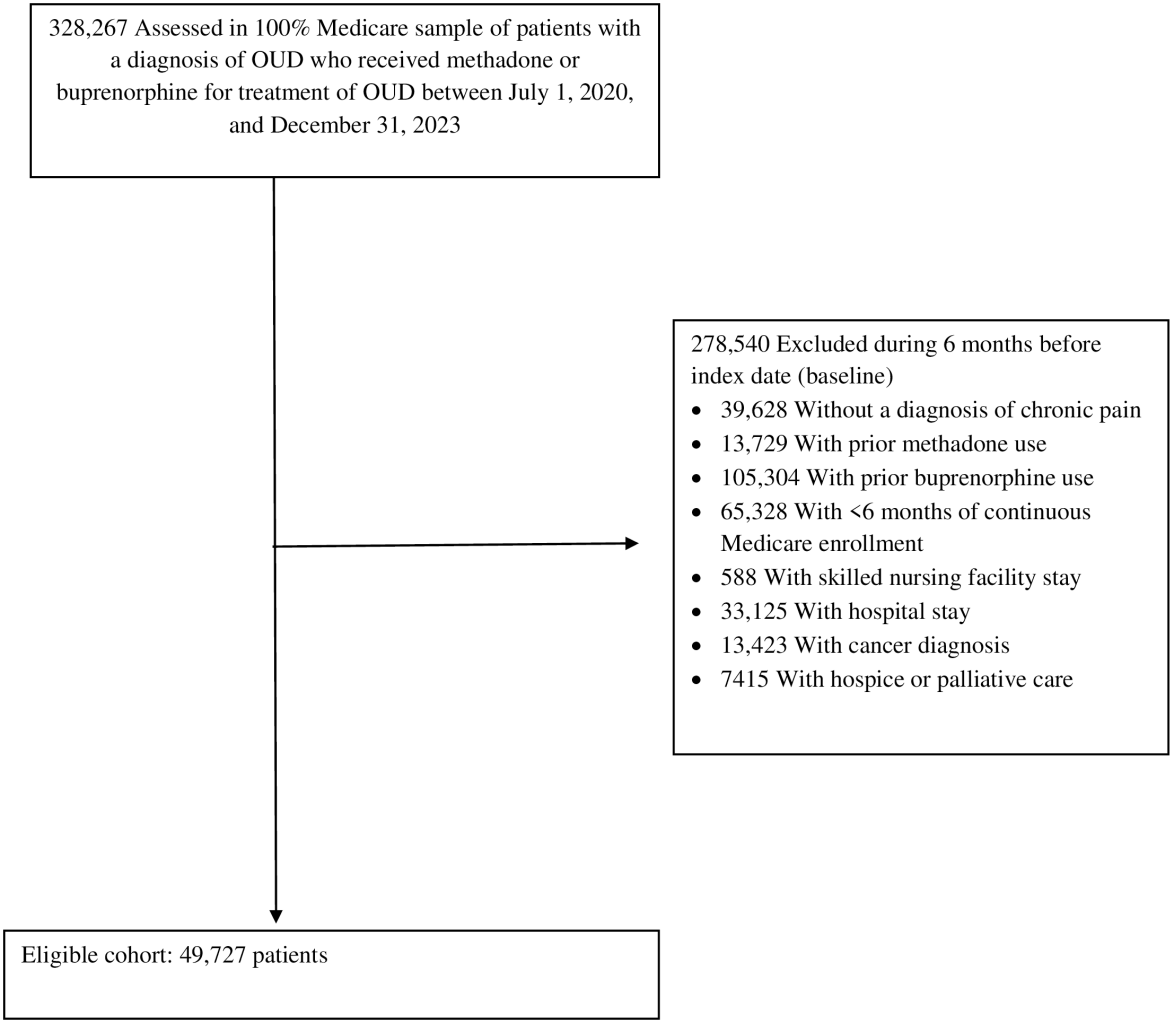
Cohort Inclusion Flowchart for the Study Sample. OUD, opioid use disorder.

In the target trial, we identified Medicare beneficiaries who initiated methadone or buprenorphine administration (index date), with no such prescription filled in the prior 6 months (baseline). The use of a 6-month baseline was to ensure the capture of treatment initiation. Because methadone and buprenorphine are covered by Medicare for pain treatment [5], to identify patients who received these medications for OUD, we required the beneficiary to (1) have a diagnosis of OUD during the 6-month baseline period, (2) initiate buprenorphine in a formulation (i.e., sublingual tablet, film, or injection) indicated for OUD [26], and (3) initiate methadone administration dispensed at OTPs only. The study sample was further limited to beneficiaries with comorbid chronic pain at baseline to reduce confounding by pain condition. Exclusion criteria included having (1) no continuous enrollment in Medicare, (2) a hospital or skilled nursing facility stay, during which Part D prescription data were unavailable to measure drug initiation and covariates, and (3) a diagnosis of cancer or palliative or hospice care because of different pain management and experience for these conditions. We then emulated the target trail using Medicare data. Figure 1 shows the sample selection details. The medications of interest and diagnostic and procedure codes for conditions and services considered in the sample selection are given in Tables A and B in S1 File.

### Treatment strategies

Treatment strategies included initiation of methadone or buprenorphine administration for OUD. Under the Medicare payment policy, methadone for OUD is dispensed only at OTPs and billed with Healthcare Common Procedure Coding System (HCPCS) codes for weekly episodes of treatment through bundled payments [6]. We identified receipt of methadone for OUD based on HCPCS codes G2067 and G2078 for weekly methadone dispensed and the date of drug dispensing through Parts B and C data. Buprenorphine for OUD can be dispensed at pharmacies under Part D or office-based clinics or OTPs under Parts B and C. We identified receipt of buprenorphine for OUD using (1) Part D data according to drug name, drug formulations indicated for OUD, days’ supply, and dispensing date; and (2) Parts B and C data according to HCPCS codes G2068, G2069, G2079, and G0533 for weekly buprenorphine dispensed and date of drug dispensing [6]. Receipts of buprenorphine in transdermal patch or buccal film only, and receipts of methadone dispensed in Part D were excluded because such use is more likely to treat pain rather than OUD. In our sample, 95.3% of buprenorphine for OUD was prescribed at office-based clinics and dispensed in pharmacies, a finding consistent with a prior study [5].

### Treatment assignment

In the intention-to-treat (ITT) analysis, randomization to treatment assignment was emulated using inverse probability of treatment weighting (IPTW) to account for observed covariates at baseline based on a conceptual framework (S1 Fig). These covariates that can be measured from Medicare data included demographic characteristics, types of Medicare plan, substance use disorders, selected clinical conditions potentially affecting pain-related outcomes, total number of comorbidities, healthcare utilization, pain management, medication use, and calendar month of treatment initiation [27]. In the per-protocol analysis, inverse probability of censoring weighting (IPCW) was also used to account for potential selection bias from additional censoring from discontinuation of methadone or buprenorphine. Predictors of treatment discontinuation measured 30 days before the end of follow-up included substance use disorders, selected clinical conditions affecting pain conditions, total number of comorbidities, pain management, and medication use. The full list of covariates, their data source, and assessment periods is provided in Table C in S1 File.

### Follow-up

In the ITT analysis, patients were followed up from the index date until the end of 1-year follow-up, death, Medicare disenrollment, or study end. In the per-protocol analysis, follow-up was additionally censored when patients discontinued or switched their treatment assigned at the index date. Discontinuation was defined as a gap in methadone or buprenorphine treatment lasting more than 28 consecutive days, the maximum allowable days’ supply of medications for OUD dispensed to treat OUD [6]. Table D in S1 File provides the distribution of censoring reasons in the overall sample and by treatment status in ITT and per-protocol analyses.

### Outcomes

Pain-related outcomes included hospitalization and emergency department (ED) visits with a primary or secondary diagnosis of a pain condition [28], assessed using *International Statistical Classification of Diseases and Related Health Problems, 10th Revision, Clinical Modification (ICD-10-CM)* diagnosis codes from Medicare Parts A, B, and C data (2 Table B in S1 File). Opioid overdose was captured from inpatient or outpatient visits with *ICD-10-CM* diagnosis codes for opioid misuse, dependence, or poisoning [28, 29]. Mortality was defined as the date of death included in the Medicare Beneficiary Summary File. For pain-related outcomes and opioid overdose, we included only patients with no prior events during the 6-month baseline and calculated incident rates of residents who experienced new events over person-days of follow-up.

### Causal contrast of interest

We estimated ITT and per-protocol effects for initiation of methadone versus buprenorphine administration. Our primary estimates of interest were the per-protocol effects of methadone versus buprenorphine when patients continued receiving the index treatment, given that discontinuation of these medications for OUD is prevalent among patients with OUD [30, 31]. Our secondary estimates of interest were ITT effects of methadone versus buprenorphine, regardless of whether patients continued to receive the index treatment during follow-up.

### Statistical analysis

In ITT analyses, we assessed baseline covariates between the methadone versus buprenorphine groups before and after IPTW, with a standardized mean difference higher than 0.100 indicating covariate imbalance [32]. IPTW was calculated as the inverse of the propensity score for methadone initiators and the inverse of 1 minus the propensity score for buprenorphine initiators. The propensity score was estimated using a logistic regression that modeled the probability of being assigned to the methadone versus buprenorphine group as the dependent variable, and baseline covariates as independent variables. Each IPTW value was truncated at the first and 99th percentiles to reduce the influence of outliers on estimates. In per-protocol analyses, we additionally calculated IPCW as the inverse of the probability of continuing the index treatment conditional on baseline covariates and follow-up covariates [33]. To stabilize the IPCW weight, we multiplied the weight by the probability of continuing the index treatment based only on baseline covariates.

In all models for estimating associations of methadone versus buprenorphine with outcomes, we incorporated IPTW for ITT associations and the product of IPTW and the stabilized IPCW for per-protocol associations. For pain-related outcomes and opioid overdose, we used a negative binomial or Poisson regression model to generate estimates of adjusted incidence rate ratios (aIRRs) and 95% confidence intervals (CIs). Days of follow-up were included as an offset variable in regression models for counts of pain-related outcomes and opioid overdose during follow-up. For all-cause mortality, we used a Cox proportional hazards regression model to estimate the hazard ratio (HR) and its 95% CI. To facilitate interpretation, for each outcome, we calculated the adjusted absolute risk of the methadone and buprenorphine groups and the adjusted risk difference (aRD) between groups. There was no missing values of baseline covariates and studied outcomes.

Several sensitivity analyses were performed to assess the robustness of the estimates by (1) additionally adjusting for censoring due to loss of follow-up via the IPCW approach, with death or Medicare disenrollment as the dependent variable and baseline covariates as independent variables; (2) stratifying the analysis by patients aged <65 versus ≥65 years; and (3) stratifying by patients with versus without Medicare-Medicaid dual eligibility given that dual-eligible patients may have unobserved medications for OUD treatment paid through Medicaid [5].

## Results

We identified 49,727 Medicare patients with a diagnosis of OUD and chronic pain at baseline who subsequently initiated methadone or buprenorphine treatment for OUD (mean [SD] age, 59.0 [11.6] years; 24,538 [49.3%] female and 25,189 [50.7%] male) (Table E in S1 File).

Of 49,727 patients, 16,174 (32.5%) initiated methadone and 33,553 (67.5%) initiated buprenorphine (Table 2). Demographics differed between beneficiaries initiating methadone versus buprenorphine in terms of sex, age, race and ethnicity, dual eligibility, region of residency, and type of Medicare enrollee. Methadone initiators were less likely to have diagnoses of substance use disorders (e.g., alcohol use disorder, stimulant use disorder, and cannabis use disorder) and clinical conditions (e.g., mental health conditions, sleep disorder, and neurodegenerative disorder) but were more likely to receive a diagnosis of infectious conditions at baseline. Pain management and medication use at baseline also differed between groups, with methadone initiators being less likely than buprenorphine initiators to receive any procedure or therapy for pain, be prescribed adjuvant analgesics, and have use of other prescription opioids, central nervous system medications, and polypharmacy (Table 2). The distribution of all measured baseline characteristics was balanced between beneficiaries initiating methadone versus buprenorphine after IPTW in ITT analyses (Table 2), and after IPTW and stabilized IPCW in per-protocol analyses (Table F in S1 File), with all standardized mean differences for characteristics <0.100 in treatment groups. It is important to note that IPTW can balance the distribution of observed covariates, not unmeasured confounders (such as pain severity), between treatment groups. In per-protocol analysis, we observed similar proportions of patients censored due to death (0.8% versus 0.6%), Medicare disenrollment (3.5% versus 5.4%), but different proportions due to discontinuing (58.6% versus 64.8%) or switching (1.1% versus 0.4%) the indexed treatment between the methadone and buprenorphine initiators (Table D in S1 File). Table G in S1 File showed baseline characteristics of the methadone and buprenorphine initiators who discontinued their indexed treatment.

**Table 2:** Characteristics of medicare patients with baseline opioid use disorder and pain who initiated methadone or buprenorphine, before and after IPTW.

Characteristic^a^	Overall cohort			IPTW cohort		
**Methadone initiators, No. (%)**	**Buprenorphine initiators, No. (%)**	**SDiff** ^**b**^	**Methadone initiators, No. (%)**	**Buprenorphine initiators, No. (%)**	**SDiff** ^**b**^
**Total No.**	**16,174**	**33,553**		**51,215**	**49,591**	
**Age, y**						
Mean (SD)	58.1 (11.0)	58.8 (11.9)				
≤64	9,590 (59.3)	21,420 (63.8)	0.094	34,685 (67.7)	31,401 (63.3)	0.093
65–74	6,051 (37.4)	9,863 (29.4)	0.171	14,796 (28.9)	15,512 (31.3)	0.052
≥75	533 (3.3)	2,270 (6.8)	0.159	1,733 (3.4)	2,678 (5.4)	0.099
**Sex**						
Male	8,970 (55.5)	16,219 (48.3)	0.143	26,666 (52.1)	25,326 (51.1)	0.020
Female	7,204 (44.5)	17,334 (51.7)		24,549 (47.9)	24,265 (48.9)	
**Race and ethnicity**						
White	9,685 (59.9)	26,234 (78.2)	0.404	37,372 (73.0)	35,859 (72.3)	0.015
Black	3,600 (22.3)	3,889 (11.6)	0.287	7,751 (15.1)	7,670 (15.5)	0.009
Other^c^	2,889 (17.9)	3,430 (10.2)	0.221	6,092 (11.9)	6,062 (12.2)	0.010
**Received dual medicare-medicaid eligibility**	13,019 (80.5)	20,749 (61.8)	0.421	34,620 (67.6)	33,683 (67.9)	0.007
**US Region**						
Northeast	5,738 (35.5)	5,523 (16.5)	0.444	11,194 (21.9)	11,101 (22.4)	0.013
Midwest	2,534 (15.7)	6,302 (18.8)	0.083	8,920 (17.4)	8,830 (17.8)	0.010
South	4,859 (30.0)	13,528 (40.3)	0.217	20,250 (39.5)	18,528 (37.4)	0.045
West	3,043 (18.8)	8,200 (24.4)	0.137	10,851 (21.2)	11,134 (22.5)	0.031
**Type of Medicare plan**						
Fee-for-service	5,722 (35.4)	17,535 (52.3)	0.253	23,794 (46.5)	21,910 (44.2)	0.046
Medicare advantage	10,452 (64.6)	16,018 (47.7)		27,421 (53.5)	27,681(55.8)	
**Substance use disorder**						
Alcohol use disorder	700 (4.3)	2,763(8.2)	0.162	4,230 (8.3)	3,492 (7.0)	0.046
Tobacco use disorder	5,827 (36.0)	11,920 (35.5)	0.011	19,490 (38.1)	17,878 (36.1)	0.042
Opioid overdose	315 (1.9)	971 (2.9)	0.062	1840 (3.6)	1,324 (2.7)	0.053
Stimulant use disorder	390 (2.4)	1931(5.8)	0.185	3,063 (6.0)	2,298 (4.6)	0.060
Cannabis use disorder	452 (2.8)	1835 (5.5)	0.135	3,036 (5.9)	2,304 (4.6)	0.057
Cocaine use disorder	561(3.5)	1,344 (4.0)	0.028	2,706 (5.3)	1955 (3.9)	0.064
Poisoned by prescription sedative medication	1,626 (10.1)	5,492 (16.4)	0.187	7,705 (15.0)	7,104 (14.3)	0.020
**Clinical condition**						
Chronic pain						
Musculoskeletal	13,989 (86.5)	31,002 (92.4)	0.193	46,366 (90.5)	44,836 (90.4)	0.004
Neuropathic	7,323 (45.3)	19,597 (58.4)	0.265	27,754 (54.2)	26,731 (53.9)	0.006
Idiopathic	4,971 (30.7)	15,202 (45.3)	0.304	20,419 (39.9)	19,920 (40.2)	0.006
Mental health disorder	8,285 (51.2)	21,934 (65.4)	0.290	31,700 (61.9)	30,151 (60.8)	0.023
Sleep disorder	2,684 (16.6)	8,518 (25.4)	0.217	11,497 (22.4)	11,117 (22.4)	0.001
Diabetes	5,565 (34.4)	10,204 (30.4)	0.085	15,754 (30.8)	15,473 (31.2)	0.010
Cardiovascular disease	4,466 (27.6)	9,990 (29.8)	0.048	14,671 (28.6)	14,333 (28.9)	0.006
Hypertension	8,654 (53.5)	18,196 (54.2)	0.015	27,050 (52.8)	26,550 (53.5)	0.014
Pulmonary condition	7,363 (45.5)	16,187 (48.2)	0.055	24,279 (47.4)	23,549 (47.5)	0.002
Kidney disease	2,913(18.0)	5,363 (16.0)	0.054	8,107 (15.8)	8,143 (16.4)	0.016
Liver disease	1,216 (7.5)	1,737 (5.2)	0.096	2,970 (5.8)	2,876 (5.8)	0.001
Gastrointestinal tract disorder	2,759 (17.1)	6,997 (20.9)	0.097	9,807 (19.1)	9,702 (19.6)	0.011
Injury	1,475 (9.1)	3,792 (11.3)	0.072	5,322 (10.4)	5,333 (10.8)	0.012
Neurodegenerative disorder	486 (3.0)	2,261(6.7)	0.174	2,708 (5.3)	2,730 (5.5)	0.010
Seizure	640 (4.0)	1,624 (4.8)	0.043	2,604 (5.1)	2,294 (4.6)	0.021
HIV infection	444 (1.3)	601 (3.7)	0.093	1,024 (2.0)	1,041 (2.1)	0.008
Hepatitis	2,134 (6.4)	2,290 (14.2)	0.153	4,609 (9.0)	4,562 (9.2)	0.008
Other infection condition^d^	4,838 (14.4)	2,885 (17.8)	0.259	7,529 (14.7)	7,587 (15.3)	0.018
Total No. of comorbidities, Mean (SD)	11.4 (6.1)	13.3 (6.9)	0.288	12.3 (11.4)	12.8 (8.2)	0.004
**Healthcare utilization**						
Any ED visit	5,861 (36.2)	13,984 (41.7)	0.118	22,345 (43.6)	20,090 (40.5)	0.063
**Pain management**						
Any procedure or therapy for pain management	2,366 (14.6)	6,945(20.7)	0.160	8,945 (17.5)	9,204 (18.6)	0.029
Use of pain medication						
Any adjuvant analgesic	8,697 (53.8)	23,785 (70.9)	0.359	33,592 (65.6)	32,334 (65.2)	0.008
Any prescription nonopioid	4,552 (28.1)	10,482 (31.2)	0.068	20,103 (39.3)	19,662 (39.6)	0.008
Use of other prescription opioids, excluding MOUDs	4,310 (26.6)	20,645(61.5)	0.750	26,150 (51.1)	24,875 (50.2)	0.018
Opioid dosage ≥20 MME/d	211 (1.3)	1,235 (3.7)	0.153	1,507 (2.9)	1,442 (2.9)	0.002
Use of long-acting opioid	178 (1.1)	1,794 (5.3)	0.242	2,412 (4.7)	1975 (4.0)	0.036
**Medication use**						
Polypharmacy	37,243 (69.6)	25,985 (77.4)	0.178	37,529 (73.3)	36,784 (74.2)	0.020
Use of other CNS medication	30,872 (53.1)	22,284 (66.4)	0.274	31,817 (62.1)	30,689 (61.9)	0.005
Use of other MOUD^e^	4,748 (9.6)	3,198 (9.5)	0.002	4,603 (9.0)	4,632 (9.3)	0.012
**Month of treatment initiation**						
January	6,001 (37.1)	2,624 (7.8)	0.749	8,297 (16.2)	7,935 (16.0)	0.006
February	808 (5.0)	2092 (6.2)	0.054	2,766 (5.4)	2,926 (5.9)	0.022
March	713 (4.4)	2,489 (7.4)	0.128	3,329 (6.5)	3,223 (6.5)	0.002
April	636 (3.9)	2,252 (6.7)	0.124	3,022 (5.9)	2,926 (5.9)	0.002
May	634(3.9)	2,165 (6.5)	0.114	2,766 (5.4)	2,827 (5.7)	0.016
June	517 (3.2)	2,406 (7.2)	0.180	3,841 (7.5)	2,975 (6.0)	0.059
July	1,463 (9.1)	3,504 (10.4)	0.047	4,814 (9.4)	5,009 (10.1)	0.024
August	1,407 (8.7)	3,459 (10.3)	0.055	4,712 (9.2)	4,910 (9.9)	0.023
September	910 (5.6)	3,310 (9.9)	0.159	4,558 (8.9)	4,314 (8.7)	0.009
October	952 (5.9)	3,403 (10.1)	0.157	4,507 (8.8)	4,414 (8.9)	0.001
November	956 (5.9)	2,979 (8.9)	0.114	4,251 (8.3)	4,017 (8.1)	0.007
December	1,177 (7.3)	2,870 (8.6)	0.047	4,404 (8.6)	4,166 (8.4)	0.009

Abbreviations: CNS, central nervous system; ED, emergency department; IPTW, inverse probability of treatment weighting; MME, morphine milligram equivalent; MOUD, medications for opioid use disorder; OTP, opioid treatment program; SDiff, standardized difference.

^a^Characteristics were measured in the 6 months before the date of initiating methadone or buprenorphine treatment.

^b^Covariates with SDiff higher than 0.100 represent meaningful differences between groups.

^c^Included Asian, Hispanic, Native American, and Pacific Islander.

^d^Included septicemia, bacterial infection (unspecified site), mycoses, viral infection, other infection (including parasitic), sexually transmitted infection (not HIV or hepatitis).

^e^Included naltrexone and naloxone.

### Pain-related outcomes

Among Medicare patients with OUD and pain at baseline, lower crude incidence rates for pain-related hospitalization (36.4 versus 47.5 per 1,000 patient-years) and ED visit (166.0 versus 177.3 per 1,000 patient-years) were observed in the methadone group compared with the buprenorphine group in the ITT analysis (Table 3). In ITT analyses with adjusted baseline covariates via IPTW, use of methadone (vs buprenorphine) was associated with reduced adjusted incidence rates of pain-related hospitalization (IRR, 0.80 (95% CI [0.75, 0.86]; *P* < .001); adjusted RD, −7.6 (95% CI [−9.8, −5.3] per 1,000 patient-years) and ED visit (IRR, 0.89 (95% CI [0.85, 0.93]; *P* < .001); adjusted RD, −17.0 (95% CI [−23.1, −11.0]) per 1,000 patient-years) (Table 3). Similar results were obtained in the per-protocol (primary) analysis, with use of methadone (vs buprenorphine) being associated with reduced adjusted incidence rates of pain-related hospitalization (IRR, 0.64 (95% CI [0.58,0.70]; *P* < .001); adjusted RD, −7.2 (95% CI [−8.8, −5.7]) per 1,000 patient-years) and ED visit (IRR, 0.87 (95% CI [0.82, 0.92]; *P* < .001); adjusted RD, −10.2 (95% CI [−14.4, −5.9]) per 1,000 patient-years) (Table 4). Sensitivity analyses additionally adjusted for censoring due to loss to follow-up yielded results consistent with the per-protocol and ITT analyses (Table G in S1 File)

**Table 3:** Intention-to-treat analysis of associations of methadone vs. buprenorphine initiation with pain-related and treatment outcomes.

	Crude estimate		Methadone vs. Buprenorphine initiators				Standardized estimate		
**Methadone initiators**	**Buprenorphine initiators**	**Methadone initiators**	**Buprenorphine initiators**	**Difference**
**Pain-related outcome**	**Incidence rate per 1,000 person-years (events/person-years), No.**		**Crude estimate** ^e^ **(95% CI)**	***P*-value** ^**f**^	**Adjusted estimate** ^e^ **(95% CI)**	***P*-value** ^**f**^	**Incidence rate per 1,000 person-years** **(95% CI)**		
Pain-related hospitalization^a^	36.4 (517/14,206), 16,174	47.5 (1306/27,475), 33,553	0.77 (0.69–0.85)	<.001	0.80 (0.75–0.86)	<.001	30.3 (28.8–31.8)	37.9 (36.2–39.6)	−7.6 (−9.8 to −5.3)
Pain-related ED visit^a,b^	166.0 (1522/9166), 10,358	177.3 (2853/16,093), 19,569	0.94 (0.88–0.99)	.04	0.89 (0.85–0.93)	<.001	134.0 (129.9–138.1)	151.0 (146.6–155.5)	−17.0 (−23.1 to −11.0)
**Treatment outcome**									
Opioid overdose^a,c^	55.8 (777/13,935), 15,859	54.9 (1465/26,708), 35,282	1.02 (0.93–1.11)	.71	1.01 (0.96–1.07)	.71	50.4 (48.4–52.3)	49.8 (47.8–51.8)	0.53 (−2.3 to 3.3)
All-cause mortality^d^	36.7 (522/14,206), 16,174	36.0 (989/27,474), 33,553	1.02 (0.91–1.13)	.77	0.96 (0.83–1.11)	.55	35.9 (34.1–37.6)	37.3 (35.5–39.1)	−1.4 (−3.9 to 1.1)

Abbreviations: ED, emergency department; CI, confidence interval

^a^Pain-related hospitalization, pain-related ED visit, and opioid overdose analyzed using a Poisson or negative binomial model with adjustment of baseline covariates (including demographics, type of Medicare plan, substance use disorders, clinical conditions, healthcare utilization, pain management, medication use, month of treatment initiation) via inverse probability of treatment weighting.

^b^Restricted to the sample with no pain-related ED visit at baseline.

^c^Restricted to the sample with no opioid overdose at baseline

^d^All-cause mortality was analyzed using a Cox hazards regression model with adjustment of baseline covariates via inverse probability of treatment weighting.

^e^Crude and adjusted estimates expressed as incidence rate ratios for pain-related hospitalization, pain-related ED visit, and opioid overdose, and expressed as a hazard ratio for all-cause mortality.

^f^P-values were generated from a negative binomial or Poisson regression model for pain-related outcomes and opioid overdose or cox proportional hazards regression model for all-cause mortality.

**Table 4:** Per-protocol analysis of associations of patients who initiated methadone vs. buprenorphine with pain-related and treatment outcomes.

	Crude estimate		Methadone vs. buprenorphine initiators				Standardized estimate		
**Methadone initiators**	**Buprenorphine initiators**	**Methadone initiators**	**Buprenorphine initiators**	**Difference**
**Pain-related outcome**	**Incidence rate per 1,000 person-years (events/person-years), No.**		**Crude estimate** ^d^ **(95% CI)**	***P*-value**	**Adjusted estimate** ^d^ **(95% CI)**	***P*-value**	**Incidence rate per 1,000 person-years** **(95% CI)**		
Pain-related hospitalization^a^	26.9 (244/9076), 16,174	35.8 (474/13,226), 33,553	0.75 (0.64–0.88)	<.001	0.64 (0.58–0.70)	<.001	12.9 (12.0–13.7)	20.1 (18.8–21.3)	−7.2 (−8.8 to −5.7)
Pain-related ED visit^a,b^	142.0 (848/5973), 10,358	143.3 (1164/8124), 19,569	0.99 (0.91–1.08)	.84	0.87 (0.82–0.92)	<.001	66.6 (64.1–69.2)	76.8 (73.4–80.0)	−10.2 (−14.4 to −5.9)
**Treatment outcome**									
Opioid overdose ^a,b^	41.6 (371/8928), 15,859	37.3 (481/12,893), 35,282	1.11 (0.97–1.28)	.12	1.02 (0.93–1.10)	.72	21.8 (20.7–23.0)	21.5 (20.1–22.9)	0.33 (−1.5 to 2.1)
All-cause mortality ^c^	16.1 (146/9076), 16,174	15.1 (199/13,226), 33,553	1.09 (0.88–1.35)	.44	1.06 (0.81–1.39)	.66	18.6 (1.71–20.2)	17.6 (1.56–19.4)	1.1 (−1.3 to 1.0)

Abbreviations: ED, emergency department; CI, confidence interval

^a^Pain-related hospitalization, pain-related ED visit, and opioid overdose analyzed using a Poisson or negative binomial model, with adjustment of baseline covariates (including demographics, type of Medicare plan, substance use disorders, clinical conditions, healthcare utilization, pain management, medication use, month of treatment initiation) via inverse probability of treatment weighting and potential selection via inverse probability weighting for censoring due to treatment discontinuation.

^b^Restricted to the sample with no corresponding outcome of interest.

^c^All-cause mortality was analyzed using a Cox hazards regression model with adjustment of baseline covariates via inverse probability of treatment weighting and potential selection via inverse probability weighting for censoring due to treatment discontinuation.

^d^Crude and adjusted estimates expressed as incidence rate ratios for pain-related hospitalization, pain-related ED visit, and opioid overdose, and expressed as a hazard ratio for all-cause mortality.

f *P*-values were generated from a negative binomial or Poisson regression model for pain-related outcomes and opioid overdose or cox proportional hazards regression model for all-cause mortality.

### Treatment outcomes

Among Medicare patients with OUD and pain at baseline, similar crude incidence rates for opioid overdose (55.8 versus 54.9 per 1,000 patient-years) and all-cause mortality (36.7 versus 36.0 per 1,000 patient-years) were observed between methadone and buprenorphine initiators in the ITT analysis (Table 3). The ITT analysis showed no difference in opioid overdose (aIRR, 1.01 (95% CI [0.96, 1.07]; *P =* .71) and all-cause mortality (adjusted HR, 0.96 (95% CI [0.83, 1.11]; *P* = .55). The per-protocol analysis yielded similar results of no difference in opioid overdose (aIRR, 1.02 (95% CI [0.93,1.10]; *P =* .72) and all-cause mortality (aIRR, 1.06 (95% CI [0.81, 1.39]; *P =* .66) between methadone and buprenorphine initiation. Results of the sensitivity with an additional IPCW weight due to loss to follow-up were consistent with those in the per-protocol and ITT analyses (Table H in S1 File).

### Results of sensitivity analyses

Analyses stratified by patients aged <65 and ≥65 years (Table I in S1 File) and their dual Medicare-Medicaid eligibility status (Table J in S1 File) yielded results consistent with the main ITT and per-protocol analyses, with reduced incidence rates of pain-related outcomes and no difference in treatment outcomes observed between initiators of methadone versus buprenorphine.

## Discussion

In this population-based cohort study of Medicare patients with OUD and chronic pain, those who initiated methadone treatment had lower risks of pain-related hospitalizations and ED visits than those who initiated buprenorphine treatment, but had no difference in opioid overdose and all-cause mortality during the 1-year follow-up in both per-protocol and ITT analyses. Consistent results were observed in sensitivity and subgroup analyses according to age and dual Medicare-Medicaid eligibility status. Our findings suggest that methadone solely administered at OTPs is associated with reduced pain-related medical encounters while showing no difference in risks of opioid overdose and all-cause mortality compared with buprenorphine primarily prescribed at office-based clinics for treatment of OUD.

Few prior clinical studies compared pain outcomes after the receipt of methadone versus buprenorphine among patients with comorbid pain and OUD [22, 34-35]. These studies, which are limited to a single clinical setting and small sample sizes, showed mixed findings, with some indicating no difference in individual pain level in the short term (6 months) [22, 34, 36], whereas one study showed reduced pain severity from baseline to 6 months in patients receiving methadone only [35]. The present study uses a national Medicare sample to detect a lower incidence rate of pain sufficiently severe to require hospitalization or ED visits in 1 year following the initiation of methadone compared with buprenorphine. In addition, the present study observed no difference in the incidence rates of opioid overdose and all-cause mortality following the initiation of methadone versus buprenorphine. Our null results derived from Medicare patients were similar to those from a population-based study conducted in Canada [37] and a systematic review [38].

The observed reduction in pain-related outcomes among patients with methadone can be explained by several reasons. First, differences in the nature of clinical settings that offer OUD treatment may explain the observed differences in pain-related outcomes between methadone and buprenorphine. In the US, Medicare patients receive methadone exclusively at OTPs, whereas buprenorphine is mostly prescribed at office-based clinics. These two clinical settings differ in that OTPs are mandated by federal law to meet various requirements, including frequent (e.g., weekly) patient assessment and monitoring, nontreatment services (e.g., counseling and psychotherapy), and comprehensive plans (e.g., drug urine test) to prevent and reduce drug diversion [18], whereas these requirements are not mandatory and are highly variable among office-based clinics that offer medications for OUD [19]. Frequent visits with intensive management at OTPs for methadone recipients may offer more clinical attention, leading to timely adjustment of methadone frequency and dose, preventing adverse drug events, such as pain-related hospitalizations and ED visits. Conversely, buprenorphine for treatment of OUD is mostly prescribed for a 30-day supply at office-based clinics [19], which may or may not closely monitor patients’ medication intake and drug-related adverse effects if they occur. Presumably, the close supervision of OUD treatment at OTPs may also result in better treatment retention and outcomes. The presumption is supported by the existing literature, which shows better treatment retention with methadone versus sublingual buprenorphine [21], but not by our present and prior studies [38] showing no difference in opioid overdose and all-cause mortality rates between the two medications for OUD.

Second, the favorable pain-related outcomes in patients with methadone may be explained by different characteristics of patient population who preferred methadone versus buprenorphine. While considering many measured patient characteristics at baseline, the present study cannot account for unmeasured confounders, such as pain and OUD severity, as suggested by baseline differences in pain treatment utilization between the methadone and buprenorphine groups. In the present study, we observed lower proportions of patients with methadone (vs buprenorphine) with receipt of pain procedure or therapy for pain management (14.6% versus 20.7%), adjuvant analgesics (53.8% versus 70.9%), and other prescription opioids (26.6% versus 61.5%) at baseline before IPTW. It is possible that patients with OUD who experience less severe pain and thus are less in need of pain management are self-selected into OTP-based treatment, leading to fewer hospitalizations and ED visits due to pain. This possibility, however, is not supported by existing literature showing greater pain severity in nonMedicare patients with methadone (vs buprenorphine) [39] or by clinical practice showing that methadone is preferred for individuals with severe OUD dependence [16]. Future studies that account for the severity of pain and OUD are warranted to confirm our findings.

Third, differential dosing practices of methadone and buprenorphine for treatment of OUD could explain observed differences in pain-related outcomes [11, 12]. Both methadone and buprenorphine are effective treatments for managing OUD and pain, but are prescribed in different dosing schedules. When used to treat OUD, a once-daily dose of methadone (60–120 mg) and buprenorphine (8–24 mg after induction) is recommended because the drugs’ ability to suppress opioid withdrawal symptoms lasts between 24 and 36 hours [40, 41]. When used to treat pain, a low or divided dose of methadone (2.5–10 mg) and buprenorphine is administered two to three times daily because the drugs’ analgesic effect lasts ~4–8 hours [42]. It is possible that patients with methadone (vs buprenorphine) had a higher mean dose in morphine milligram equivalents, leading to reduced pain-related medical encounters. This possibility, however, cannot be determined in the present study due to the lack of information on methadone dose in Medicare data. In addition, the total daily buprenorphine dose can be divided and administered multiple times daily to maximize its analgesic benefit [8], whereas splitting a once-daily methadone dose, especially early in a treatment episode, may be uncommon [43]. It is possible that patients with severe pain preferred buprenorphine treatment at the office-based setting, which is more convenient [19] and may have better access to pain management services than OTPs designed primarily to address substance use disorders, not pain [44]. Patients with severe pain likely experienced hospitalizations and ED visits due to pain. This possibility, however, cannot be verified with Medicare data that have no information on pain severity and whether the once-daily dose of buprenorphine or methadone for OUD was divided to optimize their analgesic effects.

Fourth, the difference in pharmacological properties and propensity for opioid-induced hyperalgesia between methadone and buprenorphine may also explain the differences in pain-related outcomes. Methadone offers full agonist activity at the µ-opioid receptor, which may produce sufficient analgesia, leading to better pain control and a lower risk of pain-related medical encounters, compared to a partial μ-opioid agonist, such as buprenorphine [11, 12]. On the other hand, emerging evidence suggests differential propensity for opioid-induced hyperalgesia between full and partial μ-opioid agonists [45, 46]. Compared to methadone’s full agonist activity at the μ-opioid receptor, buprenorphine's partial agonist activity and kappa-opioid receptor antagonism may provide less risk of opioid-induced hyperalgesia [45], leading to fewer pain-related medical encounters. Due to a lack of information on opioid-induced hyperalgesia in Medicare data, the present study cannot determine whether the occurrence of opioid-induced hyperalgesia during the 1-year follow-up differs between treatment groups, and if so, to what extent the differential opioid-induced hyperalgesia occurrence may modify our observed pain-related outcome differences between the methadone and buprenorphine groups. Further studies in this area are needed.

The present study provides referential data regarding pain-related and treatment outcomes of methadone versus buprenorphine for patients with comorbid chronic pain and OUD. Compared to buprenorphine prescribed primarily at office-based clinics, methadone solely administered at OTPs is associated with reduced risks of pain-related hospitalizations and ED visits while similarly controlling opioid overdose and all-cause mortality incidence rates. However, the findings of favorable pain-related outcomes in patients with methadone versus buprenorphine should be interpreted with caution because of potential differences in the underlying patient population, treatment dosing practices, pharmacological properties, and treatment practice settings, which cannot be measured in Medicare data and merit further investigations. While waiting for prospective studies to confirm our results, emerging opinion advocates the provision of methadone treatment for OUD at office-based settings in addition to OTPs [47]. A scoping review also suggests that office-based methadone may potentially enhance access to methadone treatment for patients with OUD without adversely impacting treatment outcomes [48]. It is noted that methadone has FDA black box warnings on respiratory depression, overdose, and QT-prolongation [49]. Office-based methadone, if implemented with proper recognition of the FDA warnings, may improve OUD treatment intake, which remains low, with only 18% of Medicare patients with OUD receiving any OUD treatment in 2022 [4].

This study has limitations. First, Medicare prescription drug event data provide information on prescription drugs dispensed but not consumed. Second, Medicare data may be missing medications for OUD reimbursed by Medicaid. Yet stratifying analysis according to Medicare-Medicaid dual eligibility status showed similar results, suggesting that medications for OUD, if missing under Medicare, may have minimally impacted our estimates. Third, Medicare Part D does not include prescriptions paid for through other programs (e.g., veteran benefits) nor provide information on illicit opioid use. Fourth, we could not examine any associations with dose because information on methadone dose is unavailable under Medicare bundled payments. Fifth, while adjusting for a wide range of covariates, the present study cannot fully account for unmeasured confounders, such as OUD and pain severity, physical functioning, patients’ motivation for seeking methadone versus buprenorphine treatment for OUD, or psychosocial support systems. The inability to measure these unmeasured confounders via Medicare data may lead to residual confounding and bias our estimates. Sixth, Medicare claims data have no information on individual pain intensity. We relied on pain-related medical encounters as a proxy for pain outcomes, which could miss patients who experienced pain but did not seek emergency or inpatient care. Finally, our findings are generalizable only to Medicare patients, who are predominantly older adults or individuals with disabilities, with a diagnosis of comorbid chronic pain and OUD, and receiving treatment.

This population-based cohort study of Medicare patients with comorbid chronic pain and OUD found lower incidence rates for pain-related hospitalizations and pain-related ED visits, with no difference in opioid overdose and all-cause mortality rates within 1 year following initiation of methadone compared with buprenorphine. These findings suggest that methadone solely administered at OTPs is associated with reduced pain-related outcomes while offering treatment outcomes similar to buprenorphine primarily prescribed at office-based clinics. The favorable pain-related outcomes in patients with methadone versus buprenorphine should be interpreted with caution, as the finding may reflect differences in the underlying patient population, treatment dosing practices, pharmacological properties, and treatment practice settings, which merit further investigations.

## Supporting information

S1 TARGET Checklist Transparent Reporting of Observational Studies Emulating a Target Trial (TARGET) guideline. (DOCX)

S1 Text Pre-specified analytical protocol. (DOCX)

S1 File Supplementary tables. **Table A.** Medications of interest considered in the study. **Table B**. *ICD-10-CM* or procedure codes for disease, condition, and service care considered in the study. **Table C.** Study covariates, definitions, and measurement sources and windows. **Table D.** Reasons for censoring, overall and by indexed treatment status in intention-to-treat and per-protocol analyses. **Table E.** Baseline characteristics of eligible patients with comorbid chronic pain and opioid use disorder who initiated methadone or buprenorphine. **Table F.** Baseline characteristics of the study sample who discontinued the indexed treatment for opioid use disorder. **Table G.** Standardized mean differences after applying inverse probability weighting for treatment and censoring due to loss to follow-up (mortality and medicare disenrollment) in the per-protocol analysis. **Table H**. Associations of methadone versus buprenorphine with opioid-related and treatment outcomes, adjusting for censoring due to loss to follow-up via inverse probability of censoring weighting. **Table I**. Associations of methadone versus buprenorphine use with opioid-related and treatment outcomes, stratified by patients aged <65 and ≥65 years. **Table J**. Associations of methadone versus buprenorphine use with pain-related and treatment outcomes, stratified by dual medicare-medicaid eligibility status.
(DOCX)

S1 Fig Conceptual framework for potential confounders and modifiers of the associations of methadone versus buprenorphine use with pain-related and treatment outcomes. (DOCX)

## Abbreviations

aIRRs: adjusted incidence rate ratios
aRD: adjusted risk difference
CIs: 95% confidence intervals
ED: emergency department
FDA: Food and Drug Administration
HCPCS: Healthcare Common Procedure Coding System
HRs: hazard ratios
IPCW: inverse probability of censoring weighting
IPTW: inverse probability of treatment weighting
IRRs: incidence rate ratios
ITT: intention-to-treat
OUD: opioid use disorder
OTPs: opioid treatment programs
TARGET: Transparent Reporting of Observational Studies Emulating a Target Trial.
