# Supplementary material for: Pain and treatment outcomes after initiating methadone vs buprenorphine among medicare patients with opioid use disorder and comorbid chronic pain: A target trial emulation
Source: PLoS Med. 2026 Mar 26;23(3):e1004846. doi: 10.1371/journal.pmed.1004846 (PMC13020835; doi:10.1371/journal.pmed.1004846)
Supplement: S1 Text — (DOCX) [file pmed.1004846.s002.docx]

**Study Aims:** To determine associations of methadone vs buprenorphine with pain-related and treatment outcomes among Medicare patients with opioid use disorder and chronic pain.

- Pain-related outcomes: pain-related hospitalizations and pain-related emergency department (ED) visits
- Treatment outcomes: diagnosis of fatal and nonfatal opioid overdose and all-cause mortality

**Data source:** 100% Medicare data from 2020-2023 for Fee-for-service enrollees and 2020-2022 for Medicare Advantage enrollees.

**Research design:** Retrospective cohort study with a target trial emulation (TTE) framework. The following are the 7 key elements of the TTE framework for the hypothetical trial. Plan to present these key elements in a table (e.g., Table 1) in the manuscript file.

Element 1: Eligibility criteria:

1. Initiating methadone or buprenorphine that is indicated for opioid use disorder (OUD)
   1. Methadone for OUD is only offered at opioid treatment programs under Part B bundled payment billed through HCPC codes (G2067 and G2078)
   2. Buprenorphine for OUD is offered through multiple settings, including
      1. Opioid treatment programs under Part B bundled payment billed through HCPC codes (G2068, G2069, G2079, and G0533)
      2. Office-based clinics billed through HCPCS codes (G2068, G2069, G2079, and G0533). Some patients may require buprenorphine injection at office-based clinics.
      3. Office-based clinics with prescriptions dispensed as formulations in sublingual tablets, film, or injections from pharmacies under Medicare Part D.
   3. The index date was defined as day 1 of initiating methadone or buprenorphine, with no such prescription fills dispensed within 6 months preceding the index date.
   4. Excluding receipts of buprenorphine prescribed in a transdermal patch or buccal film only in Part D, as the use is likely to treat pain, not OUD.
   5. Excluding receipts of methadone dispensed in Part D because such use is more likely to treat pain rather than OUD.
2. Having a diagnosis of OUD during the 6-month baseline period.
3. Having at least one of these three chronic pain conditions: a) musculoskeletal pain; b) neuropathic pain; or c) idiopathic pain diagnosed during the 6-month baseline period to reduce confounding by pain condition
4. Having continuously enrolled in Parts A and B for fee-for-service enrollees and Part C for Medicare Advantage enrollees during the 6-month baseline period
5. Having no hospital or skilled nursing facility stay during the 6-month baseline period (because no prescription data are available)
6. Having no cancer, hospice care, and palliative care during the 6-month baseline period (because of different pain management and experience for these conditions).

Final samples: Plant to present sample selection in a flow chart (i.e., Figure 1).

Element 2: Treatment Strategies: initiation of methadone or buprenorphine administration for OUD.

Element 3: Treatment Assignment:

1. Intention-to-treat (ITT) analysis: randomization to treatment assignment was emulated using inverse probability of treatment weighting (IPTW) to account for observed covariates at baseline
2. Per-protocol analysis: inverse probability of censoring weighting (IPCW) was also used to account for potential selection bias from additional censoring from discontinuation of methadone or buprenorphine.
   1. The IPCW is calculated as the inverse of the probability of continuing the index treatment conditional on baseline covariates and follow-up covariates. A stabilized IPCW weight, calculated as the IPCW multiplied by the probability of continuing the index treatment based only on baseline covariates, is used for the purpose of stabilizing the weight.
3. Covariates include:
   1. Demographics: age, sex, race/ethnicity, region, dual Medicare-Medicaid eligibility
   2. Medicare plan type: FFS vs MA
   3. Substance use disorders: tobacco use disorder, alcohol use disorder, stimulant use disorder, cannabis use disorder, cocaine use disorder, poisoned by prescription sedative medication, opioid overdose, measured based on their ICD-10-CM codes
   4. Clinical conditions: chronic pain type (musculoskeletal, neuropathic, or idiopathic pain), mental health disorder, sleep disorder, hypertension, diabetes, cardiovascular disease, pulmonary condition, gastrointestinal tract disease, injury, neurodegenerative disease, seizure, and other infectious conditions, all of which are measured based on HCUP CCS for ICD-10-CM.
      1. Other infectious conditions include HIV, hepatitis, septicemia, bacterial infection, mycoses, viral infection, other infections, including parasitic, and sexually transmitted infections (not HIV or hepatitis)
   5. pain management: Receipt of procedure or therapy for chronic pain management, dosage of opioids other than methadone or buprenorphine, use of long-acting opioid, use of prescription nonopioid, use of adjuvant analgesics.
   6. Medication use: Use of other CNS medication, polypharmacy, Use of other medication for opioid use disorder (naltrexone, naloxone)

Element 4: Follow-up:

1. ITT analysis: from the index date until the end of 1-year follow-up, death, Medicare disenrollment, or study end
2. Per-protocol analysis: follow-up was additionally censored when patients discontinued or switched their treatment assigned at the index date.
   1. Discontinuation was defined as a gap in methadone or buprenorphine treatment lasting more than 28 consecutive days, the maximum allowable days’ supply of medications for OUD dispensed to treat OUD.
3. Plan to present a table describing reasons for censoring during the 1-year follow-up.

Element 5: Outcomes:

1. Pain-related outcomes included hospitalization and emergency department (ED) visits with a primary or secondary diagnosis of a pain condition.
2. Treatment outcomes:
   1. Opioid overdose was captured from inpatient or outpatient visits with *ICD-10-CM* diagnosis codes.
   2. Mortality was defined as the date of death included in the Medicare Beneficiary Summary File.

Element 6: Causal contrast of interest: ITT and per-protocol analysis of methadone vs buprenorphine. Per-protocol analysis is the main analysis (because discontinuation of these medications for OUD is prevalent).

Element 7: Statistical Analysis: Our overall analytic approach includes descriptive analyses, inverse probability of treatment weighting (IPTW) to balance covariates between the methadone (study) and buprenorphine (comparison) group for the ITT analysis, and additional stabilized IPCW for per-protocol analysis. We will perform outcome models with IPTW and IPCW weights for pain-related outcomes and treatment outcomes, and sensitivity analyses. All tests are two-sided with a statistical significance of P < .05. Our analytical plan is detailed as follows:

1. Descriptive analysis:
2. A table for describing baseline characteristics of the sample before and after the IPTW, overall and by the study vs comparison group for the ITT analysis.
3. A table for describing baseline characteristics of the sample before and after applying Inverse Probability Weighting for overall and by the study vs comparison group for the per-protocol analysis.
4. Final models:
5. Pain-related outcomes and opioid overdose, we used a negative binomial or Poisson regression model to generate estimates of adjusted incidence rate ratios (aIRRs) and 95% confidence intervals (CIs). For all-cause mortality, we used a Cox proportional hazards regression model to estimate the hazard ratio (HR) and its 95% CI.
6. A table presenting associations of methadone vs. buprenorphine Initiation with pain-related and treatment outcomes in the ITT analysis.
7. A table presenting associations of methadone vs. buprenorphine Initiation with pain-related and treatment outcomes in per-protocol analysis.
8. Sensitivity analysis
9. additionally adjusting for censoring due to loss of follow-up via the IPCW approach, with death or Medicare disenrollment as the dependent variable and baseline covariates as independent variables
10. stratifying the analysis by patients aged <65 vs. ≥65 years.
11. stratifying by patients with vs without Medicare-Medicaid dual eligibility, given that dual-eligible patients may have unobserved medications for OUD treatment paid through Medicaid
12. Planned supplemental tables:

**S1 File:**

- **Table A.** Medications of Interest Considered in the Study
- **Table B**. *ICD-10-CM* or Procedure Codes for Disease, Condition, and Service Care Considered in the Study
- **Table C.** Study Covariates, Definitions, and Measurement Sources and Windows
- **Table D.** Reasons for Censoring, Overall and by Indexed Treatment Status in Intention-to-Treat and Per-Protocol Analyses
- **Table E.** Baseline Characteristics of Eligible Patients With Comorbid Chronic Pain and Opioid Use Disorder Who Initiated Methadone or Buprenorphine
- **Table F.** Standardized Mean Differences After Applying Inverse Probability Weighting for Treatment and Censoring Due to Loss to Follow-up (Mortality and Medicare Disenrollment) in the Per-Protocol Analysis
- **Table G.** Baseline Characteristics of the Study Sample Who Discontinued the Indexed Treatment for Opioid Use Disorder
- **Table H**. Associations of Methadone vs Buprenorphine with Opioid-Related and Treatment Outcomes, Adjusting for Censoring Due to Loss to Follow-Up via Inverse Probability of Censoring Weighting
- **Table I**. Associations of Methadone vs Buprenorphine Use With Opioid-Related and Treatment Outcomes, Stratified By Patients Aged <65 and ≥65 years
- **Table J**. Associations of Methadone vs Buprenorphine Use With Pain-Related and Treatment Outcomes, Stratified By Dual Medicare-Medicaid Eligibility Status

**S1 Fig.** Conceptual Framework for Potential Confounders and Modifiers of the Associations of Methadone vs Buprenorphine Use with Pain-related and Treatment Outcomes

**SAS Codes for Final Analytical ITT Analysis**

/*

counts of pain-related outcomes or opioid overdose

1. hosp_cpain: count of pain-related hospitalizations

2. ed_cpain: count of pain-related ED visit

3. OD: count of opioid overdose

key exposure: case (1: study group vs 0: comparison group)

other variables:

--log_time: log of follow-up days

--IPTW for ITT analysis

--bene_id: encrypted ID number represents each patient.

final_adv: final analytical dataset for pain-related hospitalization and ED visit outcomes (at the patient level)

final_adv_1: final analytical dataset for OD (at the resident episode level), excluding patients with OD at baseline.

*/

/*Poisson model*/

%macro adv_p (out);

/*crude*/

PROC GENMOD data=final_adv ;

CLASS bene_id case (ref='0') ;

MODEL &out=case /dist=poisson link=log offset=log_time;

ODS OUTPUT ParameterEstimates=_crude;

run;

data _crude_1;

retain Parameter Level1 RR RR_L RR_U Probz;

set _crude;

RR=round (exp (estimate), 0.01);

RR_L=round (exp (LOWERWaldCL), 0.01);

RR_U=round (exp (UPPERWaldCL), 0.01);

rename ProbChisq=Probz;

if Parameter='case';

keep RR: Parameter ProbChisq LEVEL1;

run;

/*weighted*/

PROC GENMOD data=final_adv;

CLASS bene_id case (ref='0') ;

MODEL &out=case /dist=poisson link=log offset=log_time;

WEIGHT IPTW;

ODS OUTPUT ParameterEstimates=_adj;

run;

data _adj_1;

retain Parameter Level1 RR_a RR_L_a RR_U_a Probz_a;

set _adj;

RR_a=round (exp (estimate), 0.01);

RR_L_a=round (exp (LOWERWaldCL), 0.01);

RR_U_a=round (exp (UPPERWaldCL), 0.01);

rename ProbChisq=Probz_a;

if Parameter='case';

keep RR: Parameter ProbChisq LEVEL1;

run;

proc sort data=_crude_1;by parameter level1;run;

proc sort data=_adj_1;by parameter level1;run;

data reg_&out;

merge _crude_1 _adj_1;

by parameter level1;

run;

proc datasets;delete _crude: _adj:; quit;

%mend;

%adv_p(hosp_cpain);

%adv_p(ed_cpain);

/*Negative Binomial model*/

%macro adv_nb (out);

/*crude*/

PROC GENMOD data=final_adv_1;

CLASS bene_id case (ref='0') ;

MODEL &out=case /dist=negbin link=log offset=log_time;

ODS OUTPUT ParameterEstimates=_crude;

run;

data _crude_1;

retain Parameter Level1 RR RR_L RR_U Probz;

set _crude;

RR=round (exp (estimate), 0.01);

RR_L=round (exp (LOWERWaldCL), 0.01);

RR_U=round (exp (UPPERWaldCL), 0.01);

rename ProbChisq=Probz;

if Parameter='case';

keep RR: Parameter ProbChisq LEVEL1;

run;

/*weighted*/

PROC GENMOD data=final_adv_1;

CLASS bene_id case (ref='0') ;

MODEL &out=case /dist=negbin link=log offset=log_time;

WEIGHT IPTW;

ODS OUTPUT ParameterEstimates=_adj;

run;

data _adj_1;

retain Parameter Level1 RR_a RR_L_a RR_U_a Probz_a;

set _adj;

RR_a=round (exp (estimate), 0.01);

RR_L_a=round (exp (LOWERWaldCL), 0.01);

RR_U_a=round (exp (UPPERWaldCL), 0.01);

rename ProbChisq=Probz_a;

if Parameter='case';

keep RR: Parameter ProbChisq LEVEL1;

run;

proc sort data=_crude_1;by parameter level1;run;

proc sort data=_adj_1;by parameter level1;run;

data reg_&out;

merge _crude_1 _adj_1;

by parameter level1;

run;

proc datasets;delete _crude: _adj:; quit;

%mend;

%adv_nb(OD);
