# Supplementary material for: Pain and treatment outcomes after initiating methadone vs buprenorphine among medicare patients with opioid use disorder and comorbid chronic pain: A target trial emulation
Source: PLoS Med. 2026 Mar 26;23(3):e1004846. doi: 10.1371/journal.pmed.1004846 (PMC13020835; doi:10.1371/journal.pmed.1004846)
Supplement: S1 File — Table A. Medications of interest considered in the study. Table B. ICD-10-CM or procedure codes for disease, condition, and service care considered in the study. Table C. Study covariates, definitions, and measurement sources and windows. Table D. Reasons for censoring, overall and by indexed treatment status in intention-to-treat and per-protocol analyses. Table E. Baseline characteristics of eligible patients with comorbid chronic pain and opioid use disorder who initiated methadone or buprenorphine. Table F. Baseline characteristics of the study sample who discontinued the indexed treatment for opioid use disorder. Table G. Standardized mean differences after applying inverse probability weighting for treatment and censoring due to loss to follow-up (mortality and medicare disenrollment) in the per-protocol analysis. Table H. Associations of methadone versus buprenorphine with opioid-related and treatment outcomes, adjusting for censoring due to loss to follow-up via inverse probability of censoring weighting. Table I. Associations of methadone versus buprenorphine use with opioid-related and treatment outcomes, stratified by patients aged <65 and ≥65 years. Table J. Associations of methadone versus buprenorphine use with pain-related and treatment outcomes, stratified by dual medicare-medicaid eligibility status. (DOCX) [file pmed.1004846.s003.docx]

**Supplementary Online Content**

**Table A.** Medications of Interest Considered in the Study

**Table B**. *ICD-10-CM* or Procedure Codes for Disease, Condition, and Service Care Considered in the Study

**Table C.** Study Covariates, Definitions, and Measurement Sources and Windows

**Table D.** Reasons for Censoring, Overall and by Indexed Treatment Status in Intention-to-Treat and Per-Protocol Analyses

**Table E.** Baseline Characteristics of Eligible Patients With Comorbid Chronic Pain and Opioid Use Disorder Who Initiated Methadone or Buprenorphine

**Table F.** Standardized Mean Differences After Applying Inverse Probability Weighting for Treatment and Censoring Due to Loss to Follow-up (Mortality and Medicare Disenrollment) in the Per-Protocol Analysis

**Table G.** Baseline Characteristics of the Study Sample Who Discontinued the Indexed Treatment for Opioid Use Disorder

**Table H**. Associations of Methadone vs Buprenorphine with Opioid-Related and Treatment Outcomes, Adjusting for Censoring Due to Loss to Follow-Up via Inverse Probability of Censoring Weighting

**Table I**. Associations of Methadone vs Buprenorphine Use With Opioid-Related and Treatment Outcomes, Stratified By Patients Aged <65 and ≥65 years

**Table J**. Associations of Methadone vs Buprenorphine Use With Pain-Related and Treatment Outcomes, Stratified By Dual Medicare-Medicaid Eligibility Status

**Table A.** Medications of Interest Considered in the Study

| **Medication Class** | **Individual Medication** |
| --- | --- |
| **MOUD** | Under Medicare, methadone is prescribed and dispensed from OTPs and billed through HCPCS codes: G2067 and G2078 under Medicare Part B (for fee-for-service enrollees) or Part C (for Medicare Advantage enrollees) |
|  | Under Medicare, buprenorphine is dispensed as formulations in sublingual tablets, film, or injection from a pharmacy under Medicare Part D or from OTPs or office-based clinics billed through HCPCS codes: G2068, G2069, G2079, and G0533 under Medicare Part B (for fee-for-service enrollees) or Part C (for Medicare Advantage enrollees) |
| **Other Opioids** | butorphanol, codeine, dihydrocodeine, fentanyl, hydrocodone, hydromorphone, levorphanol, meperidine, morphine, nalbuphine, opium, oxycodone, oxymorphone, pentazocine, remifentanil, sufentanil, tramadol  Excludes injectable opioids used primarily for inpatients, rectal dosage forms that are rarely used, and opioids used as cold medicines (eg, promethazine/codeine syrup) to suppress cough |
| **Nonopioid** |  |
| Analgesics and antipyretics | acetaminophen, salicylamide, sodium thiosalicylate, ziconotide |
| NSAIDs | celecoxib, diclofenac potassium/sodium, etodolac, fenoprofen, flurbiprofen, ibuprofen, indomethacin, ketoprofen, ketorolac, meclofenamate, mefenamic acid, meloxicam, nabumetone, naproxen, oxaprozin, piroxicam, sulindac, tolmetin |
| Salicylates | aspirin, diflunisal, choline magnesium trisalicylate, magnesium salicylate, and salsalate |
| **Adjuvant analgesic** |  |
| SNRI antidepressants | duloxetine, venlafaxine, desvenlafaxine, Milnacipran, levomilnacipran |
| TCA antidepressants | amitriptyline, clomipramine, imipramine, doxepin, trimipramine, amoxapine, maprotiline, nortriptyline, desipramine, protriptyline, |
| Anticonvulsants (for pain treatment) | carbamazepine, fosphenytoin, gabapentin, lacosamide, lamotrigine, levetiracetam, oxcarbazepine, phenytoin, pregabalin, topiramate, and valproate |
| Skeletal muscle relaxants | baclofen, carisoprodol, chlorzoxazone, cyclobenzaprine, dantrolene, diazepam, metaxalone, methocarbamol, orphenadrine, tizanidine |
| **Other CNS Medication** |  |
| Benzodiazepines | alprazolam, estazolam, lorazepam, oxazepam, temazepam, triazolam, midazolam, chlordiazepoxide, clobazam, clonazepam, clorazepate, diazepam, prazepam, flurazepam, quazepam |
| Antipsychotics | acetophenazine, chlorpromazine, droperidol, fluphenazine, haloperidol, loxapine, mesoridazine, molindone, perphenazine, pimozide, prochlorperazine, thioridazine, thiothixene, trifluoperazine, aripiprazole, asenapine, brexpiprazole, cariprazine, clozapine, iloperidone, fanapt, lurasidone, olanzapine, paliperidone, primavaserin, quetiapine, risperidone, ziprasidone |
| Sedative-hypnotics | buspirone, meprobamate, eszopiclone, zaleplon, zolpidem, suvorexant, hydroxyzine, diphenhydramine, ramelteon, tasimelteon, amobarbital, butabarbital, pentobarbital, secobarbital, phenobarbital, mephobarbital, chloral hydrate, lemborexant |
| Anticonvulsants (excluding those for pain treatment) | [acetazolamide](https://www.medicines.org.uk/emc/search?q=Acetazolamide), brivaracetam, cenobamate, eslicarbazepine acetate, ethadione, ethotoin, ethosuximide, ezogabine, felbamate, fenfluramine, mephenytoin, methohexital, methsuximide, magnesium sulfate, perampanel, paramethadione, phenobarbital, primidone, rufinamide, stiripentol, sultiame, tiagabine, trimethadione, vigabatrin, zonisamide |
| **Other MOUD** | Naltrexone, naloxone |

Abbreviations: CNS, central nervous system; HCPCS, Healthcare Common Procedure Coding System; NSAID, nonsteroidal anti-inflammatory; SSRI, selective serotonin reuptake inhibitor; SNRI, serotonin and norepinephrine reuptake inhibitor; CNS, central nervous system; TCA, tricyclic antidepressant; MOUDs, medications for opioid use disorder

**Table B**. *ICD-10-CM* or Procedure Codes for Disease, Condition, and Service Care Considered in the Study

| **Disease, Condition, or Service Care** | ***ICD-10-CM* or Procedure Code** | **Algorithm** |
| --- | --- | --- |
| Opioid use disorder | F11.xx | At least 1 inpatient, SNF, HHA, or carrier claim with disease code in any diagnostic position |
| **Exclusion criteria** |  |  |
| Cancer diagnosis | CCS11-CCS43 | HCUP CCS for *ICD-10-CM* |
| Hospice care | Admission date of hospice claims | At least 1 hospice claim in any diagnostic position |
| Palliative care | DX: V 66.7  Specialty code: 17 | At least 1 inpatient, SNF, HHA, HOP, carrier, or DME claim with disease code in any diagnostic position; or at least 1 inpatient, SNF, HHA, HOP with provider specialty code |
| **Pain-related outcome** |  |  |
| Pain-related hospitalization | Admission to an inpatient with a primary or secondary diagnosis of a chronic pain condition (see below) | Inpatient claims |
| Pain-related ED | Admission to ED with a primary or secondary diagnosis of a chronic pain condition (see below) | Inpatient claims |
| **Treatment outcomes** |  |  |
| Opioid overdose | T400X1-T400X5, T401X1-T401X4, T402X1-T403X5, T404X1-T404X5, T40601-T40605, T40691-T40695, excluding codes that indicate “in remission” or “subsequent encounter to identify an incident event | At least 1 inpatient, SNF, HHA, or carrier claim with disease code in any diagnostic position |
| All-cause mortality | Date of death | Medicare Beneficiary Summary File |
| **Substance use disorder** |  |  |
| Tobacco use disorder | F17.2x, O99.33x, T65.21xA, T65.22xA, T65.29xA, Z72.0 | At least 1 inpatient, SNF, HHA, HOP, carrier, or DME claim with disease code in any diagnostic position |
| Alcohol use disorder | F10.1x, F10.2x, F10.9x, G62.1, I42.6, K29.2x, K70.x, P04.3, Q86.0, T51.0XxA, Z71.4x, T51.0X1A, T51.0X2A, T51.0X3A, T51.0X4A, Z71.41, Z71.42 |  |
| Stimulant use disorder | F15.1x, F15.2x, F15.9x, T43.6x (excluding [T43.61](https://www.aapc.com/codes/icd-10-codes/T43.61)) |  |
| Cannabis use disorder | F12.1x, F12.2x, F12.9x, T40.7x |  |
| Cocaine use disorder | F14.1x, F14.2x, F14.9x, [T40.5x](https://www.aapc.com/codes/icd-10-codes/T40.5X) |  |
| Poisoned by prescription sedative medication | F13.1x, F13.2x, F13.9x |  |
| **Clinical condition** |  |  |
| Chronic pain |  |  |
| Musculoskeletal | A18.01-A18.02, A52.16, D48.1, E08.61x, E09.61x, E10.61x, E11.61x, E13.61x, M00-M02, M04.02-M04.09, M05-M19, M1A, M20.10, M21.61-M21.62, M22-M25, M32-M36, M43.2-M43.8X9, M45-M48, M49.80, M50, M51, M53, M54, M60.0-M60.2, M61-M63, M65-M67, M70-M72, M75-M77, M79, M96.1, M99.2-M99.7, N20.0, Q68.6, R25.2, R26.2, R29.8x | At least 1 inpatient, SNF, HHA, HOP, or carrier claim with disease code in any diagnostic position |
| Neuropathic | A52.15, B02 (exclude B02.1), EXX.4, EXX.610, EXX.65 (where X in “08”-“13”), E10.4, F45.42, G13.0, G13.1, G32.0, G35, G50- G52.1, G54-G59, G61.8, G61.9, G62.8, G62.9, G63-G65, G89.0, G90.0, G90.5, G95, G99.0-G99.2, M05.5, M54.13-M54.18, M54.3, M54.4, M60.8, M60.9, M79.1, M79.2, M79.7 |  |
| Idiopathic | G89, R52 |  |
| Mental disorder | Adjustment disorder (CCS 650), anxiety disorders (CCS 651), attention-deficit, conduct, and disruptive behavior disorders (CCS 652), impulse control disorders (CCS 656), mood disorders (CCS 657), personality disorders (CCS 658), schizophrenia and other psychotic disorders (CCS 659), Miscellaneous mental health disorders (CCS 670) | HCUP CCS for *ICD-10-CM* |
| Sleep disorder | F51.x, G25.9, G47.X, R06.81 | At least 1 inpatient, SNF, HHA, HOP, carrier, or DME claim with disease code in any diagnostic position |
| Diabetes | Diabetes mellitus without complication (CCS 49), diabetes mellitus with complications (CCS 50) | HCUP CCS for *ICD-10-CM* |
| Cardiovascular disease | Heart valve disorders (CCS 96), coronary atherosclerosis and other heart disease (CCS 101), pulmonary heart disease (CCS 103), cardiac dysrhythmias (CCS 106), congestive heart failure; non-hypertensive (CCS 108), acute cerebrovascular disease (CCS 109), occlusion or stenosis of precerebral arteries (CCS 110), other and ill-defined cerebrovascular disease (CCS 111), peripheral and visceral atherosclerosis (CCS 114) |  |
| Hypertension | essential hypertension (CCS 98), hypertension with complications, and secondary hypertension (CCS 99) |  |
| Pulmonary condition | Pneumonia (except that caused by tuberculosis or sexually transmitted disease) (CCS 122), acute bronchitis (CCS 125), other upper respiratory infections (CCS 126), chronic obstructive pulmonary disease and bronchiectasis (CCS 127), asthma (CCS 128), pleurisy; pneumothorax; pulmonary collapse (CCS 130), respiratory failure; insufficiency; arrest (CCS 131), other lower respiratory disease (CCS 133) |  |
| Gastrointestinal tract disorder | Gastrointestinal hemorrhage (CCS 153), other gastrointestinal disorders (CCS 155), digestive congenital anomalies (CCS 214) |  |
| Kidney disease | Nephritis; nephritis; renal sclerosis (CCS 156), acute and unspecified renal failure (CCS 157), chronic kidney disease (CCS 158), other diseases of kidney and ureters (CCS 161) |  |
| Liver disease | Liver diseases (CCS 151) |  |
| Injury | Pathological fracture (CCS 207), fracture of neck of femur (hip) (CCS 226), skull and face fractures (CCS 228), fracture of upper limb (CCS 229), fracture of lower limb (CCS 230), other fractures (CCS 231), e-codes: fall (CCS 2603) |  |
| Neurodegenerative disorder | Parkinson disease (CCS 79), other hereditary and degenerative nervous system conditions (CCS 81) |  |
| Seizure | Epilepsy; convulsions (CCS 83) |  |
| HIV infection | CCS5 |  |
| Hepatitis | CCS6 |  |
| Other infectious conditions | Septicemia (CCS2), bacterial infection (CCS3), mycoses (CCS4), viral infection (CCS7), other infections, including parasitic (CCS8), and sexually transmitted infections (not HIV or hepatitis) (CCS9) |  |
| **Procedures** |  |  |
| Medical procedures and therapies for chronic pain management | 20550-20552, 20560, 20561, 28899, 20999, 62281, 62324, 62325, 64405, 64408, 64415, 64417, 64418, 64420, 64421, 64425, 64430, 64435, 64445-64451, 64461-64463, 64505, 64510, 64517, 64520, 64530, 64620, 64632, 64660, 64999, 64450, 76881, 76882, 76942, 76999, 90901, 90912, 90913, 92506, 97001-97004, 97010, 97012, 97016, 97018, 97022, 97024, 97026, 97028, 97032-97036, 97039, 97110, 97112, 97113, 97116, 97124, 97139, 97140, 97150, 97530, 97542, 97597, 97598, 97760, 97799, 97610, 97161-97168, 97810, 97811, 97813, 97814, G0281, G0282, G0283, G0329, 0019T, 98940, 98941, 98942, 98943 | At least 1 inpatient, SNF, HHA, HOP, carrier, or DME claim with disease code |

Abbreviations: CCS, Clinical Classification Software; DME, durable medical equipment; ED, emergency department; HCUP, Healthcare Cost and Utilization Project; HHA, home health agency; HOP, hospital outpatient; HIV, human immunodeficiency virus; *ICD-10-CM*, *International Classification of Diseases, Tenth Revision, Clinical Modification*; SNF, skilled nursing facility.

**Table C**. Study Covariates, Definitions, and Measurement Sources and Windows

| **Covariate** | **Measurement** | **Measurement window** | **Data source** |
| --- | --- | --- | --- |
| **Demographic characteristic** |  |  |  |
| Age | Continuous variable | On index date | Medicare Beneficiary Summary File |
| Sex | Male or female | On index date | Medicare Beneficiary Summary File |
| Race and ethnicity | Measured based on the Medicare-Research Triangle Institute race code and grouped into 3 groups: Black, White, and other (including Asian, Hispanic, Native American, and Pacific Islander) | On index date | Medicare Beneficiary Summary File |
| US geographic region | Northeast, Midwest, West, and South | On index date | Medicare Beneficiary Summary File |
| Receipt of Medicare-Medicaid dual eligibility | Receipt of dual eligibility was defined based on monthly indicator of dual eligibility. Patients who have at least 6 months of receiving dual eligibility are considered dual eligibles; otherwise, not. | 6 months before index date | Medicare Beneficiary Summary File |
| **Medicare plan type** | FFS vs MA enrollees.  FFS enrollees were defined as having both Parts A and B plans for at least 4 of 6 months before the index date, based on monthly Part A or Part B entitlement indicator (code: 3 or C).  MA enrollees were defined as having an MA plan for at least 4 of 6 months before the index date, based on the MA enrollment indicator (code: 1, 2, A, B, or C).  Note: <1% of the study sample enrolled in both FFS and MA plans in a single month; if they had at least 4 months of enrollment in both plans, they were grouped as FFS enrollees. | 6 months before index date | Medicare Beneficiary Summary File |
| **Substance use disorders**^1^ |  |  |  |
| Tobacco use disorder | Presence or absence of diagnosis | 6 months before the index date | Medicare Parts A, B, and C |
| Alcohol use disorder | Presence or absence of diagnosis | 6 months before the index date | Medicare Parts A, B, and C |
| Opioid overdose | Presence or absence of diagnosis | 6 months before the index date | Medicare Parts A, B, and C |
| Stimulant use disorder | Presence or absence of diagnosis | 6 months before the index date | Medicare Parts A, B, and C |
| Cannabis use disorder | Presence or absence of diagnosis | 6 months before the index date | Medicare Parts A, B, and C |
| Cocaine use disorder | Presence or absence of diagnosis | 6 months before the index date | Medicare Parts A, B, and C |
| Poisoned by prescription sedative medication | Presence or absence of diagnosis | 6 months before the index date | Medicare Parts A, B, and C |
| **Clinical condition**^1^ |  |  |  |
| Chronic pain | Presence or absence of diagnosis | 6 months before the index date | Medicare Parts A, B, and C |
| Musculoskeletal | Presence or absence of diagnosis | 6 months before the index date | Medicare Parts A, B, and C |
| Neuropathic | Presence or absence of diagnosis | 6 months before the index date | Medicare Parts A, B, and C |
| Idiopathic | Presence or absence of diagnosis | 6 months before the index date | Medicare Parts A, B, and C |
| Mental health disorder | Presence or absence of diagnosis | 6 months before the index date | Medicare Parts A, B, and C |
| Sleep disorder | Presence or absence of diagnosis | 6 months before the index date | Medicare Parts A, B, and C |
| Hypertension | Presence or absence of diagnosis | 6 months before the index date | Medicare Parts A, B, and C |
| Diabetes | Presence or absence of diagnosis | 6 months before the index date | Medicare Parts A, B, and C |
| Cardiovascular disease | Presence or absence of diagnosis | 6 months before the index date | Medicare Parts A, B, and C |
| Pulmonary condition | Presence or absence of diagnosis | 6 months before the index date | Medicare Parts A, B, and C |
| Gastrointestinal tract disease | Presence or absence of diagnosis | 6 months before the index date | Medicare Parts A, B, and C |
| Injury | Presence or absence of diagnosis | 6 months before the index date | Medicare Parts A, B, and C |
| Neurodegenerative disease | Presence or absence of diagnosis | 6 months before the index date | Medicare Parts A, B, and C |
| Seizure | Presence or absence of diagnosis | 6 months before the index date | Medicare Parts A, B, and C |
| HIV infection | Presence or absence of diagnosis | 6 months before the index date | Medicare Parts A, B, and C |
| Hepatitis | Presence or absence of diagnosis | 6 months before the index date | Medicare Parts A, B, and C |
| Other infectious conditions | Presence or absence of diagnosis | 6 months before the index date | Medicare Parts A, B, and C |
| Total number of comorbidities | Sum of the number of AHRQ CCS categories | 6 months before the index date | Medicare Parts A, B, and C |
| **Pain management**^1^ |  |  |  |
| Receipt of procedure or therapy for chronic pain management | Presence or absence of CPT codes | 6 months before the index date | Medicare Parts A, B, and C |
| Opioid dosage | Classified as <20 or ≥20 MME/day | 6 months before the index date | Medicare Part D |
| Use of long-acting opioid | Yes vs. no (list of long-acting) | 6 months before the index date | Medicare Part D |
| Use of prescription nonopioid | Yes vs. no | 6 months before the index date | Medicare Part D |
| Use of adjuvant analgesic | Yes vs. no | 6 months before the index date | Medicare Part D |
| **Medication use**^1^ |  |  |  |
| Use of other CNS medication | Yes vs. no | 6 months before the index date | Medicare Part D |
| Polypharmacy | Yes vs no  Polypharmacy was defined as use of ≥5 dispensed generic drugs (excluding studied MOUD) | 6 months before the index date | Medicare Part D |
| Use of other MOUD | Yes vs. no | 6 months before the index date | Medicare Parts B, C, and D |
| Calendar month of treatment initiation | Categorical variable | On the index date | Medicare Parts B, C, and D |

Abbreviations: AHRQ, Agency of Healthcare Research and Quality; CCS, Clinical Classification Software; CNS, central nervous system; CPT, current procedural terminology; FFS, fee-for-service; MA, Medicare Advantage; MME, morphine milligram equivalents; MOUD, medication for opioid use disorder.

^1^ also measured 30 days before the end of follow-up

**Table D.** Reasons for Censoring, Overall and by Indexed Treatment Status in Intention-to-Treat and Per-Protocol analyses

|  | Censoring reason, No. (%) of Participants | | | | |
| --- | --- | --- | --- | --- | --- |
|  | Discontinuation due to death | Discontinued Medicare enrollment | Study end ^a^ | Discontinued indexed treatment | Switched  indexed treatment ^b^ |
| Overall sample (n=49,727) | 1511 (3.0) | 6222 (12.5) | 15022 (30.2) | NA | NA |
| *By Indexed Treatment Status* | ***Intention-to-treat analysis*** | | | | |
| Methadone (n=16,174) | 522 (3.2) | 1387 (8.6) | 7575 (46.8) | NA | NA |
| Buprenorphine (n=33,553) | 989 (2.9) | 4835 (14.4) | 7447(22.2) | NA | NA |
| *By Indexed Treatment Status* | ***Per-protocol analysis*** | | | | |
| Methadone (n=16,174) | 125 (0.8) | 558 (3.5) | 3596 (22.2) | 9481 (58.6) | 171(1.06) |
| Buprenorphine (n=33,553) | 184 (0.6) | 1822 (5.4) | 3172 (9.5) | 21745 (64.8) | 129 (0.40) |

Abbreviation: NA, not applicable.

^a^ Study end was December 31, 2022, for Medicaid Advantage enrollees and December 31, 2023, for fee-for-service enrollees.

^b^ Switching drug was measured within 14 days following the discontinuation of the indexed treatment.

**Table E.** Baseline Characteristics of Eligible Patients With Comorbid Chronic Pain and Opioid Use Disorder Who Initiated Methadone or Buprenorphine

| **Characteristic** | **Overall Sample** | | |
| --- | --- | --- | --- |
|  | **No.** | | **%** |
| **Total No.** | 49,727 | 100 | |
|  |  |  | |
| **Age, y** |  |  | |
| Mean (SD) | 59.0 | 11.6 | |
| ≤64 | 31,010 | 62.4 | |
| 65-74 | 15,914 | 32.0 | |
| ≥75 | 2803 | 5.6 | |
| **Sex** |  |  | |
| Male | 25,189 | 50.7 | |
| Female | 24,538 | 49.3 | |
| **Race and ethnicity** |  |  | |
| White | 35,919 | 72.2 | |
| Black | 7489 | 15.1 | |
| Other | 6319 | 12.7 | |
| **Received dual Medicare-Medicaid eligibility** | 337,68 | 67.9 | |
| **US Region** |  |  | |
| Northeast | 11,261 | 22.6 | |
| Midwest | 8836 | 17.8 | |
| South | 18,387 | 37.0 | |
| West | 11,243 | 22.6 | |
| **Type of Medicare plan** |  |  | |
| Fee-for-service | 21,740 | 43.7 | |
| Medicare advantage | 27,987 | 56.3 | |
| **Substance use disorder** |  |  | |
| Alcohol use disorder | 3463 | 7.0 | |
| Tobacco use disorder | 17,747 | 35.7 | |
| Opioid overdose | 1286 | 2.6 | |
| Stimulant use disorder | 2280 | 4.6 | |
| Cannabis use disorder | 2287 | 4.6 | |
| Cocaine use disorder | 1905 | 3.8 | |
| Poisoned by prescription sedative medication | 7118 | 14.3 | |
| **Clinical condition** |  |  | |
| Chronic pain |  |  | |
| Musculoskeletal | 44,991 | 90.5 | |
| Neuropathic | 26,920 | 54.1 | |
| Idiopathic | 20,173 | 40.6 | |
| Mental health disorder | 30,219 | 60.8 | |
| Sleep disorders | 11,202 | 22.5 | |
| Diabetes | 15,769 | 31.7 | |
| Cardiovascular disease | 14,456 | 29.1 | |
| Hypertension | 26,850 | 54.0 | |
| Pulmonary condition | 23,550 | 47.4 | |
| Kidney disease | 8276 | 16.6 | |
| Liver disease | 2953 | 5.9 | |
| Gastrointestinal tract disorder | 9756 | 19.6 | |
| Injury | 5267 | 10.6 | |
| Neurodegenerative disorder | 2747 | 5.5 | |
| Seizure | 2264 | 4.6 | |
| HIV infection | 1045 | 2.1 | |
| Hepatitis | 4424 | 8.9 | |
| Other infectious condition | 7723 | 15.5 | |
| Total No. of comorbidities, mean (SD) | 12.7 | 6.7 | |
| **Healthcare utilization** |  |  | |
| Any ED visit | 19,800 | 39.8 | |
| **Pain management** |  |  | |
| Any procedure or therapy for pain management | 9311 | 18.7 | |
| Use of pain medication |  |  | |
| Any adjuvant analgesic | 32,482 | 65.3 | |
| Any prescription nonopioid | 15,034 | 30.2 | |
| Use of other prescription opioids, excluding MOUD | 24,955 | 50.2 | |
| Opioid dosage ≥20 MME/d | 1972 | 2.9 | |
| Use of long-acting opioid | 1972 | 4.0 | |
| **Medication use** |  |  | |
| Polypharmacy | 37,243 | 74.9 | |
| Use of other CNS medication | 30,872 | 62.1 | |
| Use of other MOUD | 4748 | 9.5 | |
| **Month of indexed treatment** |  |  | |
| January | 8625 | 17.3 | |
| February | 2900 | 5.8 | |
| March | 3202 | 6.4 | |
| April | 2888 | 5.8 | |
| May | 2799 | 5.6 | |
| June | 2923 | 5.9 | |
| July | 4967 | 10.0 | |
| August | 4866 | 9.8 | |
| September | 4220 | 8.5 | |
| October | 4355 | 8.8 | |
| November | 3935 | 7.9 | |
| December | 4047 | 8.1 | |

Abbreviations: CNS, central nervous system; ED, emergency department; MME, morphine milligram equivalent; MOUD, medication for opioid use disorder; SD, standard deviation.

**Table F.** Standardized Mean Differences After Applying Inverse Probability Weighting for Treatment and Censoring Due to Loss to Follow-up (Mortality and Medicare Disenrollment) in the Per-Protocol Analysis

| **Characteristic** ^a^ | **Methadone Initiator, No. (%)** | **Buprenorphine Initiator, No. (%)** | **SDiff ^b^** |
| --- | --- | --- | --- |
| **Total No.** | **54,915** | **54,879** |  |
| **Age, y** |  |  |  |
| Mean (SD) | 58.1 (12.3) | 58.8 (15.1) |  |
| ≤64 | 36,738 (66.9) | 34,448 (62.8) | 0.087 |
| 65-74 | 16,143 (29.4) | 17,266 (31.5) | 0.045 |
| ≥75 | 2034 (3.7) | 3165 (5.8) | 0.097 |
| **Sex** |  |  |  |
| Male | 28,773 (52.4) | 27,717 (50.5) | 0.038 |
| Female | 26,142 (47.6) | 27,162 (49.5) |  |
| **Race and ethnicity** |  |  |  |
| White | 39,294 (71.6) | 39,715 (72.4) | 0.018 |
| Black | 9067 (16.5) | 8480 (15.5) | 0.029 |
| Other ^c^ | 6553 (11.9) | 6684 (12.2) | 0.008 |
| **Received dual Medicare-Medicaid eligibility** | 37,760 (68.8) | 36,989 (67.4) | 0.029 |
| **US Region** |  |  |  |
| Northeast | 12,073 (22.0) | 11,679 (21.3) | 0.017 |
| Midwest | 9981(18.2) | 9735 (17.7) | 0.011 |
| South | 21,288 (38.8) | 20,523 (37.4) | 0.028 |
| West | 115,72 (21.1) | 12,942 (23.6) | 0.060 |
| **Type of Medicare plan** |  |  |  |
| Fee-for-service | 25,474 (46.4) | 24,261 (44.2) | 0.044 |
| Medicare advantage | 29,441 (53.6) | 29,618 (54.0) |  |
| **Substance use disorder** |  |  |  |
| Alcohol use disorder | 4215 (7.7) | 3952 (7.2) | 0.018 |
| Tobacco use disorder | 20,972 (38.2) | 19,727 (35.9) | 0.047 |
| Opioid overdose | 1769 (3.2) | 1520 (2.8) | 0.027 |
| Stimulant use disorder | 3014 (5.5) | 2588 (4.7) | 0.035 |
| Cannabis use disorder | 2866 (5.2) | 2577 (4.7) | 0.024 |
| Cocaine use disorder | 2632 (4.8) | 2202(4.0) | 0.038 |
| Poisoned by prescription sedative medication | 8164 (14.9) | 8009 (14.6) | 0.008 |
| **Clinical condition** |  |  |  |
| Chronic pain |  |  |  |
| Musculoskeletal | 49,883 (90.8) | 49,843 (90.8) | 0.001 |
| Neuropathic | 29,946 (54.5) | 30,112 (54.9) | 0.007 |
| Idiopathic | 22,180 (40.4) | 22,646 (41.3) | 0.017 |
| Mental health disorder | 33,963 (61.8) | 33,628 (61.3) | 0.012 |
| Sleep disorders | 12,203 (22.2) | 12,636 (23.0) | 0.019 |
| Diabetes | 17,237 (31.4) | 17,228 (31.4) | 0.000 |
| Cardiovascular disease | 15,898 (29.0) | 16,390 (29.9) | 0.020 |
| Hypertension | 29,365 (53.5) | 29,708 (54.1) | 0.013 |
| Pulmonary condition | 26,258 (47.8) | 26,419 (48.1) | 0.007 |
| Kidney disease | 8862 (16.1) | 9243 (16.8) | 0.019 |
| Liver disease | 3270.6 (6.0) | 3235 (5.9) | 0.003 |
| Gastrointestinal tract disorder | 10,559 (19.2) | 11,106 (20.2) | 0.025 |
| Fall | 5760 (10.5) | 6211 (11.3) | 0.027 |
| Neurodegenerative disorder | 2898 (5.3) | 3160 (5.8) | 0.021 |
| Seizure | 2765 (5.0) | 2579 (4.7) | 0.016 |
| HIV infection | 1208 (2.2) | 1152 (2.1) | 0.014 |
| Hepatitis | 5766 (10.5) | 4445 (8.1) | 0.084 |
| Other infectious condition | 7908 (14.4) | 8726 (15.9) | 0.044 |
| Total No. of comorbidities, mean (SD) | 12.7 (12.3) | 13.0 (8.8) | 0.031 |
| **Healthcare utilization** |  |  |  |
| Any ED visit | 23,597 (43.0) | 22,725 (41.4) | 0.032 |
| **Pain management** |  |  |  |
| Any procedure or therapy for pain management | 9741 (17.7) | 10,706 (19.5) | 0.046 |
| Use of pain medication |  |  |  |
| Any adjuvant analgesic | 36,040 (65.6) | 36,181 (65.9) | 0.006 |
| Any prescription nonopioid | 22,008 (40.1) | 21,523 (39.2) | 0.018 |
| Use of other prescription opioids, excluding MOUDs | 29,032 (52.9) | 28,928 (52.7) | 0.003 |
| Opioid dosage ≥20 MME/d | 1718 (3.1) | 1684 (3.1) | 0.003 |
| Use of long-acting opioids | 2459 (4.5) | 2316 (4.2) | 0.013 |
| **Medication use** |  |  |  |
| Polypharmacy | 40,748 (74.2) | 41,074 (74.8) | 0.015 |
| Use of other CNS medication | 34,145 (62.2) | 34,260 (62.4) | 0.005 |
| Use of other MOUD ^d^ | 5056 (9.2) | 5300 (9.7) | 0.015 |

Abbreviations: CNS, central nervous system; ED, emergency department; IPTW, inverse probability of treatment weighting; MME, morphine milligram equivalent; MOUD, medications for opioid use disorder; SD, standard deviation; SDiff, standardized difference.

^a^ Characteristics were measured in the 6 months before the date of initiating methadone or buprenorphine treatment. Alos included calendar month of treatment initiation.

^b^ Covariates with SDiff higher than 0.100 represent meaningful differences between groups.

^c^ Included Asian, Hispanic, Native American, and Pacific Islander.

^d^ Included naltrexone and naloxone.

**Table G.** Baseline Characteristics of Study Sample Who Discontinued the Indexed Treatment for Opioid Use Disorder

| **Characteristic** | **Patients who discontinued Buprenorphine** | **Patients who discontinued Methadone** |
| --- | --- | --- |
|  | **No. (%)** | **No. (%)** |
| **Total No.** | 21,745 (100) | 9481 (100) |
| **Age, y** |  |  |
| Mean (SD) | 59.0 (12.3) | 59.5 (11.0) |
| ≤64 | 13,585 (62.5) | 5509 (58.1) |
| 65-74 | 6472 (29.8) | 3652 (38.5) |
| ≥75 | 1688 (7.8) | 320 (3.4) |
| **Sex** |  |  |
| Male | 10,463 (48.1) | 5299 (55.9) |
| Female | 11,282 (51.9) | 4182 (44.1) |
| **Race and ethnicity** |  |  |
| White | 16,307 (75.0) | 5454 (57.5) |
| Black | 3037 (14.0) | 2345 (14.7) |
| Other | 2401 (11.0) | 1682 (17.7) |
| **Received dual Medicare-Medicaid eligibility** | 13,535 (62.2) | 7659 (80.8) |
| **US Region** |  |  |
| Northeast | 3340 (15.4) | 3011 (31.8) |
| Midwest | 4099 (18.9) | 1680 (17.7) |
| South | 8508 (39.1) | 2945 (31.1) |
| West | 5798 (26.7) | 1845 (19.5) |
| **Type of Medicare plan** |  |  |
| Fee-for-service | 10,493 (48.3) | 3106 (32.8) |
| Medicare advantage | 11,252 (51.8) | 6375 (67.2) |
| **Substance use disorder** |  |  |
| Alcohol use disorder | 1869 (8.6) | 447 (4.7) |
| Tobacco use disorder | 7615 (35.0) | 3511 (37.0) |
| Opioid overdose | 686 (3.2) | 209 (2.2) |
| Stimulant use disorder | 1410 (6.5) | 228 (2.4) |
| Cannabis use disorder | 1221 (5.6) | 270 (2.9) |
| Cocaine use disorder | 981 (4.5) | 355 (3.7) |
| Poisoned by prescription sedative medication | 3602 (16.6) | 931 (9.8) |
| **Clinical condition** |  |  |
| Chronic pain |  |  |
| Musculoskeletal | 20147 (92.7) | 8229 (86.8) |
| Neuropathic | 12816 (58.9) | 4298 (45.3) |
| Idiopathic | 9996 (46.0) | 2926 (30.9) |
| Mental health disorder | 14255 (65.6) | 4854 (51.2) |
| Sleep disorders | 5678 (26.1) | 1545 (16.3) |
| Diabetes | 6687 (30.8) | 3264 (34.4) |
| Cardiovascular disease | 6718 (30.9) | 2719 (28.7) |
| Hypertension | 11868 (54.6) | 5165 (54.5) |
| Pulmonary condition | 10676 (49.1) | 4401 (46.4) |
| Kidney disease | 3715 (17.1) | 1692 (17.9) |
| Liver disease | 1172 (5.4) | 744 (7.9) |
| Gastrointestinal tract disorder | 4709 (21.7) | 1609 (17.0) |
| Injury | 2624 (12.1) | 892 (9.4) |
| Neurodegenerative disorder | 1523 (7.0) | 277 (2.9) |
| Seizure | 1074 (4.9) | 403 (4.3) |
| Infectious condition | 4556 (21.0) | 2940 (31.0) |
| Total No. of comorbidities, mean (SD) | 13.7 (7.1) | 11.6 (6.2) |
| **Healthcare utilization** |  |  |
| Any ED visit | 9580 (44.1) | 3539 (37.3) |
| **Pain management** |  |  |
| Any procedure or therapy for pain management | 4721 (21.7) | 1393 (14.7) |
| Use of pain medication |  |  |
| Any adjuvant analgesic | 15241 (70.1) | 4980 (52.5) |
| Any prescription nonopioid | 8315 (38.2) | 3351 (35.3) |
| Use of other prescription opioids, excluding MOUD | 13599 (62.5) | 2580 (27.2) |
| Opioid dosage ≥20 MME/d | 798 (3.7) | 116 (1.2) |
| Use of long-acting opioid | 1171 (5.4) | 99 (1.0) |
| **Medication use** |  |  |
| Polypharmacy | 16809 (77.3) | 6552 (69.1) |
| Use of other CNS medication | 14386 (66.2) | 4966 (52.4) |
| Use of other MOUD | 2120 (9.8) | 854 (9.0) |
| **Days of treatment in 1-year follow-up**, mean (SD) | 72.1 (84) | 130.6 (119.0) |
| **Month of treatment initiation** |  |  |
| January | 1750 (8.1) | 3014 (31.8) |
| February | 1463 (6.7) | 471 (5.0) |
| March | 1720 (7.9) | 442 (4.7) |
| April | 1463 (6.7) | 396 (4.2) |
| May | 1428 (6.6) | 411 (4.3) |
| June | 1540 (7.1) | 313 (3.3) |
| July | 2271 (10.4) | 928 (9.8) |
| August | 2263 (10.4) | 887 (9.4) |
| September | 2138 (9.8) | 578 (6.1) |
| October | 2203 (10.1) | 608 (6.4) |
| November | 1854 (8.5) | 621 (6.6) |
| December | 1652 (7.6) | 812 (8.6) |

Abbreviations: CNS, central nervous system; ED, emergency department; MME, morphine milligram equivalent; MOUD, medication for opioid use disorder; SD, standard deviation.

**Table H**. Associations of Methadone vs Buprenorphine with Opioid-Related and Treatment Outcomes, Adjusting for Censoring Due to Loss to Follow-Up via Inverse Probability of Censoring Weighting

|  | **Initiation of Methadone vs. Buprenorphine** | | | |
| --- | --- | --- | --- | --- |
|  | ***Intention-to-treat analysis*** ^d^ | | ***Per-protocol analysis*** ^d^ | |
|  | **Adjusted IRR**  **(95% CI)** | ***P* value** | **Adjusted IRR**  **(95% CI)** | ***P* value** |
| **Pain-related outcome** ^a^ |  |  |  |  |
| Pain-related hospitalization ^a^ | 0.78 (0.74-0.83) | <.001 | 0.64 (0.59-0.71) | <.001 |
| Pain-related ED visit ^a,b^ | 0.88 (0.85-0.91) | <.001 | 0.88 (0.83-0.93) | <.001 |
| **Treatment outcome** |  |  |  |  |
| Opioid overdose ^a,c^ | 1.02 (0.97-1.07) | .55 | 1.07 (0.98-1.16) | .13 |

Abbreviations: ED, emergency department; CI, confidence interval. IRR, incidence rate ratio.

^a^ Pain-related hospitalizations, pain-related ED visits, and opioid overdose were analyzed using a Poisson or negative binomial model.

^b^ Restricted to the sample with no pain-related ED visit at baseline.

^c^ Restricted to the sample with no opioid overdose at baseline.

^d^ In intention-to-treat analysis, estimates adjusted for baseline covariates via inverse probability of treatment weighting. In per-protocol analysis, estimates adjusted for baseline covariates via inverse probability of treatment weighting and selection bias via inverse probability weighting for censoring due to treatment discontinuation.

**Table I.** Associations of Methadone vs Buprenorphine Use With Pain-Related and Treatment Outcomes, Stratified By Patients Aged <65 and ≥65 years

|  | **Methadone vs Buprenorphine Initiation** | | | | | | | | | |
| --- | --- | --- | --- | --- | --- | --- | --- | --- | --- | --- |
|  | **Patients aged <65 years** ^b^ | | | | | **Patients** **aged ≥65 years** ^b^ | | | | |
| **Outcome** ^a^ | **n** | **Crude estimate** ^e^  **(95% CI)** | **P-value** ^f^ | **Adjusted estimate** ^e^  **(95% CI)** | **P-value** ^f^ | **n** | **Crude estimate** ^e^  **(95% CI)** | **P-value** ^f^ | **Adjusted estimate** ^e^  **(95% CI)** | **P-value** ^f^ |
| *Intention-to-treat analysis* ^g^ | | | | | | | | | | |
| Pain-related hospitalization ^a^ | 21420 | 0.88 (0.77-0.99) | 0.048 | 0.89 (0.82-0.97) | 0.006 | 12133 | 0.61 (0.51-0.72) | <.001 | 0.57 (0.51-0.64) | <.001 |
| Pain-related ED visit ^a,b^ | 11599 | 0.91 (0.84-0.99) | 0.03 | 0.90 (0.85-0.95) | <.001 | 7970 | 0.98 (0.89-1.09) | 0.76 | 0.80 (0.75-0.86) | <.001 |
| Opioid overdose ^a,c^ | 20812 | 1.15 (0.92-1.43) | 0.21 | 1.22 (0.89-1.66) | 0.22 | 11770 | 1.33 (1.05-1.68) | 0.02 | 1.12 (0.88-1.44) | 0.37 |
| All-cause mortality ^d^ | 21420 | 0.97 (0.84-1.23) | 0.71 | 0.95 (0.77-1.18) | 0.66 | 12133 | 1.01 (0.86-1.17) | 0.92 | 0.98 (0.78-1.22) | 0.87 |
| *Per-protocol analysis* ^g^ | | | | | | | | | | |
| Pain-related hospitalization ^a^ | 21420 | 0.87 (0.72-1.06) | 0.17 | 0.77 (0.69-0.87) | <.001 | 12133 | 0.58 (0.45-0.75) | <.001 | 0.47 (0.40-0.55) | <.001 |
| Pain-related ED visit ^a,b^ | 11599 | 0.93 (0.83-1.04) | 0.22 | 0.87(0.81-0.94) | <.001 | 7970 | 1.11 (0.96-1.29) | 0.15 | 0.90 (0.82-0.99) | .02 |
| Opioid overdose ^a,c^ | 20812 | 0.95 (0.79-1.14) | 0.60 | 0.93 (0.84-1.04) | 0.21 | 11770 | 0.71 (0.46-1.08) | 0.11 | 0.69 (0.45-1.06) | 0.19 |
| All-cause mortality ^d^ | 21420 | 1.02 (0.75-1.37) | 0.90 | 1.01 (0.70-1.46) | 0.96 | 12133 | 1.09 (0.80-1.48) | 0.59 | 1.24 (0.81-1.89) | 0.32 |

Abbreviations: ED, emergency department; CI, confidence interval

^a^ Pain-related hospitalizations, pain-related ED visits, and opioid overdose were analyzed using a Poisson or negative binomial model.

^b^ Restricted to the sample with no pain-related ed visit at baseline.

^c^ Restricted to the sample with no opioid overdose at baseline.

^d^ All-cause mortality was analyzed using a Cox hazards regression model.

^e^ Crude and adjusted estimates expressed as incidence rate ratios for pain-related hospitalizations, pain-related ED visits, and opioid overdose, and expressed as a hazard ratio for all-cause mortality.

^f^ P-values were generated from a negative binomial or Poisson regression model for pain-related outcomes and opioid overdose or cox proportional hazards regression model for all-cause mortality.

^g^ In intention-to-treat analysis, estimates were adjusted for baseline covariates via inverse probability of treatment weighting. In per-protocol analysis, estimates were adjusted for baseline covariates via inverse probability of treatment weighting and selection bias via inverse probability weighting for censoring due to treatment discontinuation.

**Table J.** Associations of Methadone vs Buprenorphine Use With Pain-Related and Treatment Outcomes, Stratified By Dual Medicare-Medicaid Eligibility Status

|  | **Methadone vs Buprenorphine Use** | | | | | | | | | | |
| --- | --- | --- | --- | --- | --- | --- | --- | --- | --- | --- | --- |
|  | **Patients With Medicare-Medicaid Dual Eligibility** | | | | | | **Patients Without Medicare-Medicaid Dual Eligibility** | | | | |
| **Outcome** ^a^ | **n** | **Crude estimate** ^e^  **(95% CI)** | **P-value** ^f^ | **Adjusted estimate** ^e^  **(95% CI)** | **P-value** ^f^ | **n** | | **Crude estimate** ^e^  **(95% CI)** | **P-value** ^f^ | **Adjusted estimate** ^e^  **(95% CI)** | **P-value** ^f^ |
| *ITT analysis* ^g^ | | | | | | | | | | | |
| Pain-related hospitalization ^a^ | 33,768 | 0.77 (0.68-0.87) | <.001 | 0.77 (0.68-0.87) | <.001 | 15,959 | | 0.76 (0.61-0.94) | 0.01 | 0.66 (0.58-0.75) | <.001 |
| Pain-related ED visit ^a,b^ | 19,009 | 0.87 (0.81-0.93) | <.001 | 0.87 (0.81-0.93) | <.001 | 10,918 | | 0.87 (0.75-0.99) | 0.047 | 0.84 (0.78-0.91) | <.001 |
| Opioid overdose ^a,c^ | 32,853 | 0.88 (0.75-1.03) | 0.10 | 0.88 (0.75-1.03) | 0.10 | 15,588 | | 1.15 (0.92-1.43) | 0.21 | 1.22 (0.89-1.66) | 0.22 |
| All-cause mortality ^d^ | 33,768 | 1.00 (0.88-1.13) | 0.99 | 0.88 (0.75-1.02) | 0.09 | 15,959 | | 0.99 (0.78-1.24) | 0.91 | 1.15 (0.84-1.57) | 0.37 |
| *Per-protocol analysis* ^g^ | | | | | | | | | | | |
| Pain-related hospitalization ^a^ | 33,768 | 0.77 (0.64-0.92) | 0.005 | 0.70 (0.62-0.78) | <.001 | 15,959 | | 0.75 (0.54-1.02) | 0.07 | 0.47 (0.39-0.56) | <.001 |
| Pain-related ED visit ^a,b^ | 19,009 | 0.93 (0.84-1.03) | 0.17 | 0.88 (0.82-0.95) | <.001 | 10,918 | | 0.90 (0.74-1.09) | 0.28 | 0.83 (0.74-0.93) | <.001 |
| Opioid overdose ^a,c^ | 32,853 | 1.07 (0.92-1.24) | 0.41 | 1.00 (0.91-1.11) | 0.96 | 15,588 | | 0.92 (0.66-1.28) | 0.63 | 1.17 (0.99-1.36) | 0.05 |
| All-cause mortality ^e^ | 33,768 | 0.99 (0.77-1.27) | 0.94 | 1.00 (0.73-1.37) | 0.99 | 15,959 | | 1.35 (0.88-2.08) | 0.17 | 1.24 (0.81-1.89) | 0.32 |

Abbreviations: ED, emergency department; CI, confidence interval

^a^ Pain-related hospitalizations and ED visits and opioid overdose were analyzed using a Poisson or negative binomial model

^b^ Restricted to the sample with no pain-related ed visit at baseline.

^c^ Restricted to the sample with no opioid overdose at baseline.

^d^ All-cause mortality was analyzed using a Cox hazards regression model.

^e^ Crude and adjusted estimates expressed as incidence rate ratios for pain-related hospitalizations, pain-related ED visits, and opioid overdose, and expressed as a hazard ratio for all-cause mortality.

^f^ P-values were generated from a negative binomial or Poisson regression model for pain-related outcomes and opioid overdose or cox proportional hazards regression model for all-cause mortality.

^g^ In intention-to-treat analysis, estimates were adjusted for baseline covariates via inverse probability of treatment weighting. In per-protocol analysis, estimates were adjusted for baseline covariates via inverse probability of treatment weighting and selection bias via inverse probability weighting for censoring due to treatment discontinuation.
