# Supplementary material for: Pain and treatment outcomes after initiating methadone vs buprenorphine among medicare patients with opioid use disorder and comorbid chronic pain: A target trial emulation
Source: PLoS Med. 2026 Mar 26;23(3):e1004846. doi: 10.1371/journal.pmed.1004846 (PMC13020835; doi:10.1371/journal.pmed.1004846)
Supplement: S1 Fig — (DOCX) [file pmed.1004846.s004.docx]

**Potential confounders**

- **Demographics**
- **Substance use disorders**
- **Clinical conditions**
- **Health care utilization**
- **Pain management**
- **Medication use**
- Health status
- Physical functioning
- Pain chronicity
- Disease severity
- Pain severity
- OUD severity
- MOUD dosing
- Treatment settings
- Others
- Motivation for treatment
- Psychosocial supporting system
- Distance to OUD treatment settings
- Non-medical opioid use

**Potential modifiers**

- Opioid-induced hyperalgesia
- Retention of OUD treatment
- Whether OUD treatment dosing was modified

**Key Exposure**

Methadone vs buprenorphine for treatment of OUD

**Outcomes**

- Hospitalizations due to pain

- ED visits due to pain

- Opioid overdose

- All-cause mortality

**S1 Fig**. **Conceptual framework for potential confounders and modifiers of the associations of methadone vs buprenorphine use with pain-related and treatment outcomes.** ED, emergency department; OUD, opioid use disorder; MOUD, medications for opioid use disorder. We adjusted for potential confounders that are bolded in our analysis.
